# Single alloy nanoparticle x-ray imaging during a catalytic reaction

**DOI:** 10.1126/sciadv.abh0757

**Published:** 2021-10-01

**Authors:** Young Yong Kim, Thomas F. Keller, Tiago J. Goncalves, Manuel Abuin, Henning Runge, Luca Gelisio, Jerome Carnis, Vedran Vonk, Philipp N. Plessow, Ivan A. Vartaniants, Andreas Stierle

**Affiliations:** 1 Deutsches Elektronen-Synchrotron (DESY), D-22607 Hamburg, Germany.; 2 University of Hamburg, Physics Department, D-20355 Hamburg, Germany.; 3 Institute of Catalysis Research and Technology, Karlsruhe Institute of Technology, D-76344 Eggenstein-Leopoldshafen, Germany.; 4 National Research Nuclear University MEPhI, Moscow 115409, Russia.

## Abstract

The imaging of active nanoparticles represents a milestone in decoding heterogeneous catalysts’ dynamics. We report the facet-resolved, surface strain state of a single PtRh alloy nanoparticle on SrTiO_3_ determined by coherent x-ray diffraction imaging under catalytic reaction conditions. Density functional theory calculations allow us to correlate the facet surface strain state to its reaction environment–dependent chemical composition. We find that the initially Pt-terminated nanoparticle surface gets Rh-enriched under CO oxidation reaction conditions. The local composition is facet orientation dependent, and the Rh enrichment is nonreversible under subsequent CO reduction. Tracking facet-resolved strain and composition under operando conditions is crucial for a rational design of more efficient heterogeneous catalysts with tailored activity, selectivity, and lifetime.

## Teaser

We have imaged a single alloy catalyst nanoparticle at work, resolving its facet surface composition.

## INTRODUCTION

Heterogeneous catalysts play a decisive role for today’s and future industrial-scale energy production, conversion, and storage, as well as for exhaust gas cleaning in environmental applications (*1*, *2*). Further on, they are involved in more than 80% of all chemical production processes (*3*). At the nanoscale, heterogeneous catalysts are composed of oxide-supported, active nanoparticles of varying size, shape, and composition (*4*). Under operating conditions, the catalyst is exposed to reactive gas mixtures at atmospheric or higher pressures and elevated temperatures, rendering it a rather intricate, dynamical system. The structural complexity together with harsh reaction conditions often hampers an atomic-scale understanding of catalytic reactions, which is at the heart of any rational design of future more efficient catalysts with tailored activity, selectivity, and lifetime (*2*). To overcome these limitations, high-resolution imaging techniques are required, compatible with realistic reaction conditions. Transmission electron microscopy has made substantial progress in the past years in the investigation of nanoparticles under ambient pressure catalytic reaction conditions (*5*–*8*). In addition, coherent x-ray diffraction imaging (CXDI), among other x-ray imaging techniques, has proven to be a very powerful method for the structural characterization of nanoscale objects with nanometer resolution *(**9*–*12**)*. It allows the investigation of the three-dimensional (3D) crystalline electron density and strain field of a single nano-object, as demonstrated for experiments under oxidizing, reducing, and reaction conditions (*13*–*18*). However, there is a lack of operando studies including structural characterization at the single-nanoparticle level of a working catalyst.

Alloy-based three-way catalysts for exhaust gas cleaning containing PtRh nanoparticles were reported to exhibit increased activity due to synergistic electronic effects (*19*). Their near-surface composition is expected to vary for different gas surroundings, affecting the activity (*8*, *19*, *20*). Here, we demonstrate that the surface of an active, single PtRh alloy catalyst nanoparticle can be imaged using x-rays under operando catalytic flow conditions during the prototypical CO oxidation reaction. We resolve the nanoparticle size, shape, and the facet orientation–dependent evolution of the surface and bulk strain field while simultaneously probing the catalytic activity. A rigorous comparison with density functional theory (DFT) calculations allows us to correlate the facet surface strain with its compositional evolution and energetics.

## RESULTS AND DISCUSSION

**Fig. 1.**
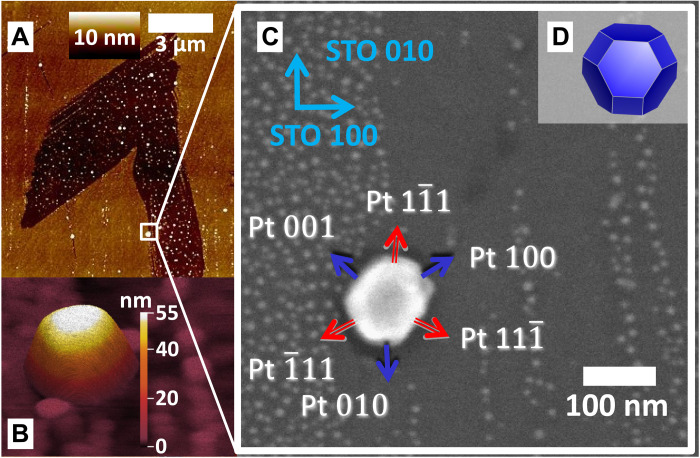
Sample architecture. (**A**) Overview topographic AFM image containing the nanoparticle investigated during the CXDI experiment. (**B**) High-resolution AFM image of the PtRh nanoparticle under investigation and smaller nanoparticles in the vicinity. (**C**) SEM zoom of the squared region marked in (A) together with nanoparticle facet and substrate directions. Blue arrows indicate <100> type facets (red arrows: <111> type facets). (**D**) Wulff-Kashiew construction of the nanoparticle equilibrium shape based on DFT surface energy calculations.

PtRh nanoparticles were prepared by co-deposition onto a SrTiO_3_ (STO) (001) substrate as described in Materials and Methods and the Supplementary Materials, with a final annealing step at 1473 K to achieve equilibrium particle shape. Substrate defects, such as step edges, promote the growth of PtRh nanoparticles with diameters around 100 nm [see atomic force microscopy (AFM) image in Fig. 1A and fig. S1].

**Fig. 2.**
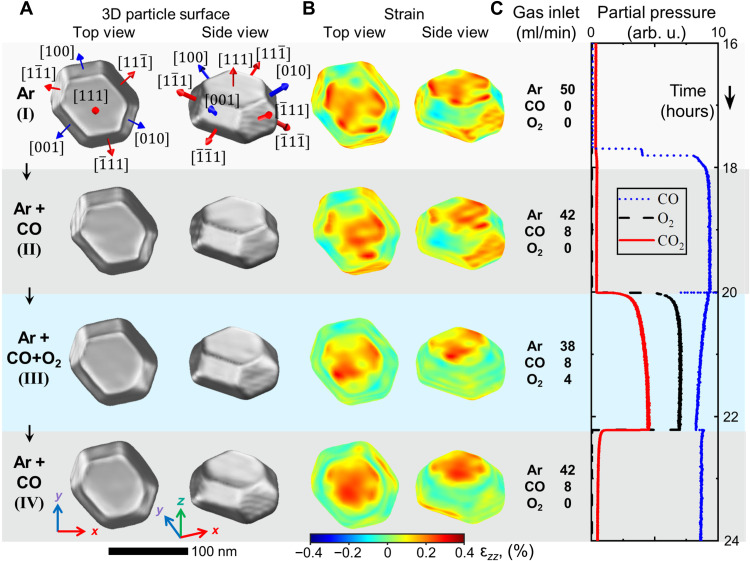
Nanoparticle shape and surface strain for different gas conditions. (**A**) Top and side views of the reconstructed nanoparticle and (**B**) strain field ϵ*_zz_* at the nanoparticle surface for gas conditions (I to IV). The position of the surface is defined as a cut at 55% of the reconstructed crystalline electron density from its maximum value. (**C**) Inlet gas composition and mass spectrometer signal during the experiment: CO (blue), O_2_ (black), and CO_2_ (red).

The CXDI catalysis experiment was performed at beamline ID01 at the European Synchrotron Radiation Facility (ESRF), Grenoble (France) using a nanofocused, monochromatic x-ray beam. The experiment was carried out at *T* = 700 K and 100 mbar reactor pressure. The following gas conditions were applied to vary between reducing to active conditions: (I) pure Ar flow at 50 ml/min; (II) CO at 8 ml/min and Ar at 42 ml/min; (III) CO at 8 ml/min, O_2_ at 4 ml/min, and Ar at 38 ml/min; and (IV) CO at 8 ml/min and Ar at 42 ml/min (see table S1). Under stoichiometric reaction conditions (III), the ensemble activity of the catalyst was proven by a high CO_2_ production (see Fig. 2C).

We selected one specific nanoparticle for in-depth characterization, which we tracked by markers during the x-ray experiment (see fig. S2). It is shown in the high-resolution AFM image in Fig. 1B and the scanning electron microscopy (SEM) image in Fig. 1C. The nanoparticle has a composition of Pt_60_Rh_40_, as determined by energy-dispersive x-ray analysis, which is consistent with the lattice parameter determined by Bragg diffraction (see fig. S3, Materials and Methods, and the Supplementary Materials). The bulk of the nanoparticle exhibits a face-centered cubic (fcc) structure, with statistical distribution of Pt and Rh atoms (*20*, *21*). The 111 Bragg reflection with scattering vector perpendicular to the top (111) surface of the nanoparticle was probed. The 2D diffraction patterns were collected during rocking scans on an Eiger2M detector (fig. S4). They were converted to reciprocal space to obtain the 3D diffraction pattern from the investigated PtRh nanoparticle (fig. S5).

The 3D nanoparticle crystalline electron density and the local strain component ϵ*_zz_* in the direction of the scattering vector were determined by phase retrieval from the 3D diffraction pattern. The voxel size of our reconstruction given by the dimensions of probed reciprocal space was about 3 nm by 3 nm by 2 nm *x*-, *y*-, and *z*-directions (see Materials and Methods and the Supplementary Materials for details). In Fig. 2A and fig. S6, the 3D nanoparticle shape is presented for gas conditions I to IV, as obtained from the reconstructed electron density. The reconstructed nanoparticle is 55 nm high, 95 nm wide, and 120 nm long, in very good agreement with SEM and AFM results (Fig. 1C and fig. S7). The facet angles can be precisely determined from cuts through the electron density (fig. S8), resulting in an exclusively low index termination of the nanoparticle by three <100> and eight <111> type facets, as indicated in Fig. 2A. The nanoparticle shape under pure Ar flow represents the as-grown situation and is in good agreement with the DFT-based Wulff-Kashiew construction (*22*) for a Pt_50_Rh_50_ nanoparticle on TiO_2_-terminated STO (001), as shown in Fig. 1D and fig. S18.

**Fig. 3.**
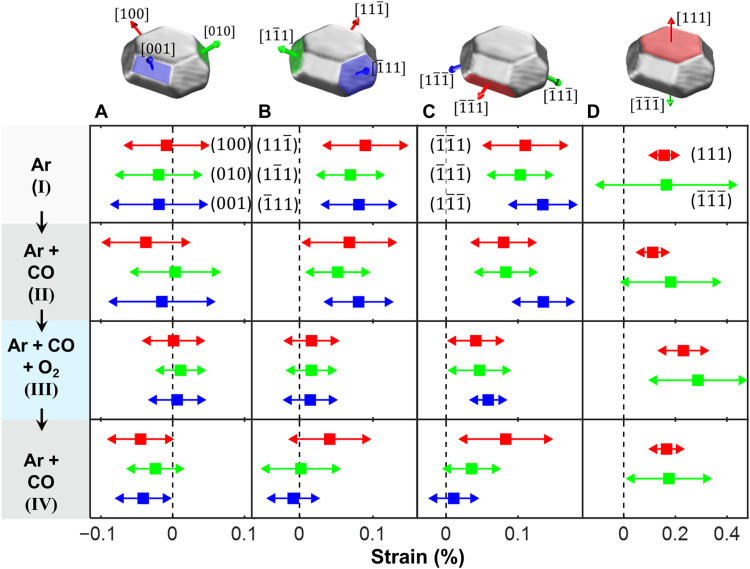
Facet-resolved strain as a function of the reaction conditions. (**A**) Top <100> type side facets, (**B**) top <111> type side facets, (**C**) bottom <111> type side facets, and (**D**) top (111) and bottom ($\bar{1}\bar{1}\bar{1}$) facet. Mean strain values, squares; SD and standard deviation (SD), lines with arrows. The colors of the facets in (A) to (D) correspond to the color of the experimental strain values in the plots respective below.

We now focus on the correlation of the PtRh nanoparticle facet surface strain evolution and their compositional change under catalytic reaction conditions. The reconstructed, facet-resolved strain field ϵ*_zz_* at the nanoparticle surface is presented in Fig. 2B. Figure 3 shows the facet-resolved average surface strain and the strain distribution for the different reaction conditions (see also fig. S11). We make the interesting observation that under Ar flow conditions (I), the <111> type facets exhibit an average outward relaxation, whereas the <100> type facets are nearly strain free. All side facets exhibit a strain distribution width of ~0.1%. The top (111) facet strain distribution is much smaller than the bottom ($\bar{1}\bar{1}\bar{1}$) facet, originating from the interfacial lattice mismatch. A similar situation is observed under Ar/CO flow conditions (II), in line with the weak interaction of CO with the nanoparticle surface under the experimental conditions (see Materials and Methods and the Supplementary Materials theory part). The strain field in the nanoparticle bulk is presented in fig. S8 and movies S1 to S4. The bulk lattice appears to be relaxed under Ar and Ar/CO conditions (the strain is close to zero; see cuts in figs. S8 and S10), with the exception of a region with negative strain close to the top (111) facet in the middle of the nanoparticle, which may be attributed to a slight subsurface Rh enrichment (*23*).

**Fig. 4.**
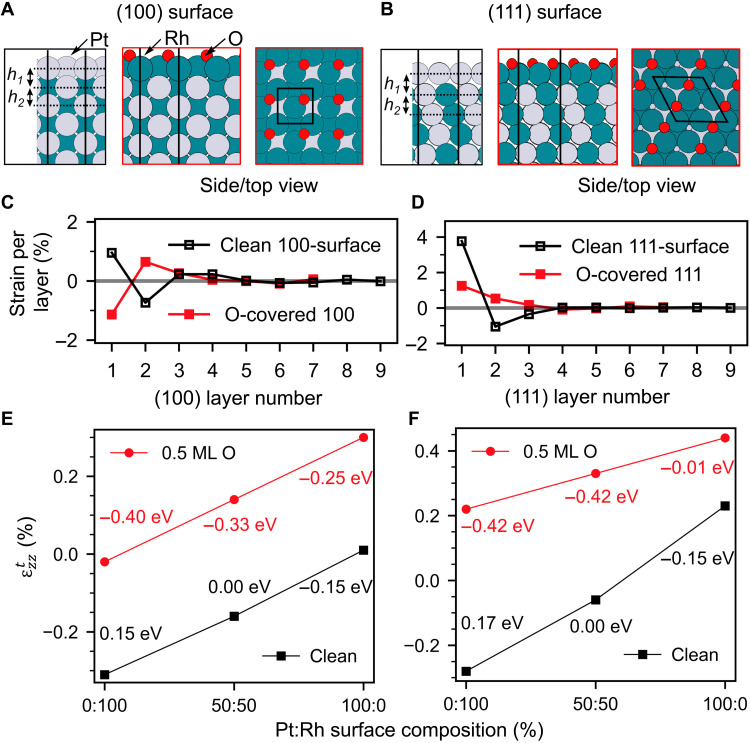
Surface composition and oxygen coverage–dependent calculated surface strain. (**A** and **B**) Side and top views of the clean and oxygen-covered (100) and (111) surfaces. (**C** and **D**) DFT surface strain profiles for the clean and 0.5 monolayer (ML) oxygen-covered (100) and (111) surface. (**E** and **F**) Trends in $\epsilon_{\mathrm{zz}}^{t}$ for the (100) and (111) surface as a function of the top layer Pt-Rh composition together with the surface energy difference per atom relative to the stoichiometric surface. Black squares, clean surface; red circles, 0.5 monolayer oxygen-covered surface.

To elucidate the origin of the anisotropic facet surface strain pattern, we calculated a large library of structures by DFT using the Perdew, Burke, Ernzerhof (PBE) functional with dispersion corrections (*24*). We determined the most stable atomic structure and composition of the nanoparticle <100> and <111> type facets (details are presented in Materials and Methods and the Supplementary Materials). Different terminations were studied by varying the composition of the outer two layers. Figure 4 shows the most stable surface structures (Fig. 4, A and B), the surface orientation, and adsorbate-dependent layer-resolved strain profiles for the most stable structures (Fig. 4, C and D). The layer-resolved strain values were averaged over the experimental voxel size (2 nm in the *z* direction) and the theoretical strain component $\epsilon_{\mathrm{zz}}^{t}$in the direction of the scattering vector was calculated according to Eq. 1 (see tables S4 and S5). To be able to compare with the experimental ϵ*_zz_* values of the side facets, the theoretical $\epsilon_{\mathrm{zz}}^{t}$ values have to be transformed to $\epsilon_{\mathrm{zz}}^{t*}=\epsilon_{\mathrm{zz}}^{t}{\text{cos}}^{2}\alpha$, where α is the angle between the facet and the substrate (α = 54° for <100> type side facets and α = 70° for <111> type side facets). The theoretical results are robust against the choice of the slab size and the functional (see tables S6 and S7). Here, we assume that the atoms mainly relax perpendicular to the facets, which is justified for larger facets. Figure 4 (E and F) shows $\epsilon_{\mathrm{zz}}^{t}$ trends for the clean and oxygen-covered (100) and (111) surfaces: At higher Rh concentrations in the topmost layer, $\epsilon_{\mathrm{zz}}^{t}$ gets more negative, in line with the 3.2% smaller atomic radius of Rh as compared to Pt. In the absence of adsorbates after deposition, in Ar atmosphere or Ar/CO mixture, we expect no significant CO coverage under our experimental conditions (see Materials and Methods and the Supplementary Materials). A 100% Pt surface termination is energetically most favorable for both (100) and (111) type facets, in agreement with previous investigations (*25*–*27*). The theoretical segregation-induced surface relaxation pattern given in Fig. 4 (C and D) exhibits distinct differences for the 111 and 100 surfaces: For the 111 surface, a more pronounced outward relaxation of the first Pt layer is observed. The calculations predict a positive value for $\epsilon_{\mathrm{zz}}^{t}$ of +0.23% for the top (111) facet, a $\epsilon_{\mathrm{zz}}^{t*}$value of +0.03% for the top <111> type side facets, and a value of $\epsilon_{\mathrm{zz}}^{t*}=$ +0.01% for the <100> type side facets. Overall, we find good agreement with the experimental observations (see Fig. 3).

In our experiments, the bottom <111> type side facets exhibit more positive strain values as compared to the top <111> side facets, suggesting a stronger influence of the substrate or additional finite facet size–induced strain effects. These effects were not included in the DFT calculations, which predict identical mean strain values for all <111> type side facets. The comparison between theoretical and experimental results demonstrates that the CXDI surface strain pattern is directly connected to the surface composition–induced relaxation state of the outmost surface layers of the nanoparticle. The comparison of the relaxation trends for <100> and <111> type facets provides evidence that under pure Ar and mixed Ar/CO flow, the whole nanoparticle surface is Pt terminated, which is also energetically most favorable under these conditions according to our calculations.

Next, we will discuss the evolution of the surface and bulk strain pattern under CO oxidation reaction condition (III). The activity of the catalyst is evident from the emerging CO_2_ mass spectrometry signal in Fig. 2C. The decreasing CO signal indicates self-activation during operation close to mass transfer limitations (*20*). Under reaction conditions, oxygen is dissociating on the Pt-terminated nanoparticle surface (*28*), and the higher affinity of oxygen to Rh as compared to Pt induces Rh surface segregation (*20*, *29*). We observe that under reaction conditions (third row in Figs. 2 and 3), strain is released over all nanoparticle side facets. This is most pronounced for the <111> type top side facets, with ϵ*_zz_* values close to zero. The strain in the <111> type bottom side facets is less reduced. For the (111) top facet, a strain reversal to more positive values is observed. The average interfacial strain slightly increased under these conditions. Another observation is the reduction of the strain distribution width under reaction conditions for the top <100> and <111> type facets to about half of the initial value, which may be related to a smoothening of the nanoparticle facets. We also observe that a rounding of the corners at the nanoparticle top takes place under reaction conditions, pointing to a reshaping at the top of the nanoparticle. This provides more active step sites, especially at the edge between the (111) and (010) side facet (see Fig. 2A, fig. S9, and movie S3) (*5*, *30*). The strain in the nanoparticle bulk is observed to fully relax also in regions closer to the nanoparticle facets, and the region of negative strain close to the top 111 facet in the middle of the nanoparticle disappeared, compatible with Rh surface segregation and concentration equilibration.

Figure 4 (E and F) shows the theoretical $\epsilon_{\mathrm{zz}}^{t}$ trends for 0.5 monolayer oxygen coverage as a function of the top most atomic layer composition. This represents the highest chemisorbed oxygen coverage to be expected under partially mass transfer–limited reaction conditions. In addition, for the oxygen adsorption structures, $\epsilon_{\mathrm{zz}}^{t}$gets more negative with increasing Rh concentration, but it is overall systematically shifted to more positive values. For both orientations, a 100% Rh surface composition is energetically more favorable in the presence of oxygen. O-Rh-O trilayer surface oxide formation is not compatible with our data (average strain, >3%; see table S8) (*20*, *31*).

The theoretical predictions rationalize our experimental observations: For the <100> type facets, ϵ*_zz_* of 0.01% is observed experimentally (compared to$\epsilon_{\mathrm{zz}}^{t*}=$−0.01%, according to our calculations for the most stable structure with 100% Rh termination and oxygen on bridge site or a slightly less stable structure with $\epsilon_{\mathrm{zz}}^{t*}=$ +0.02% for oxygen in hollow sites). In this case, the oxygen adsorption–induced increase in average strain is nearly fully compensated by the change in surface termination from 100% Pt to ≈100% Rh. Other energetically less favorable surface compositions at 0.5 monolayer oxygen coverage give rise to theoretical $\epsilon_{\mathrm{zz}}^{t*}$ values that deviate by at least 0.04% from our experimental results (see table S4).

For the oxygen-covered (111) type facets, the calculations predict a 100% Rh and stoichiometric surface composition to be energetically degenerate but more favorable than 100% Pt surface concentration (see Fig. 4F). The experimental ϵ*_zz_* values of the <111> type side facets (0.02 to 0.05%) are compatible with a Rh surface composition of ~50%. The top 111 facet exhibits an average strain of 0.22%, pointing also to a 100% Rh composition.

In the next step of the reaction sequence, the oxygen flow was set to zero and the CO flow was kept constant. A breakdown in the CO_2_ production rate was visible in the mass spectrometer signal in Fig. 2C [condition (IV)], accompanied by an increase in the CO signal due to a lifting of mass transfer limitations. Under CO-rich conditions, all oxygen-containing surface phases on the nanoparticle facets get reduced (*20*, *32*, *33*). The observed surface strain state of the nanoparticles, however, does not fully return to the previous situation under the same condition (II) (see Fig. 3, bottom row). This points to an irreversible process that took place on the facet surfaces after complete oxygen reduction: We suggest that metallic Rh is still present at the surface because of the reduced driving force for Rh subsurface segregation at 700 K (*23*, *25*). The <100> type facets exhibit a slightly negative experimental strain ϵ*_zz_* of −0.03%, which is compatible with a Pt_75_Rh_25_ surface composition according to our calculations [see Fig. 4E and table S4 ($\epsilon_{\mathrm{zz}}^{t*}=-0.05\%$ for Pt_50_Rh_50_ to +0.01% for 100% Pt in the topmost layer)]. The strain state of the ($1\bar{1}1$) and ($\bar{1}11$) top side facets is also slightly reduced as compared to reaction conditions to values around zero. This is compatible with a surface composition slightly overstoichiometric in Pt (see Fig. 4F). The ($11\bar{1}$) top side facet and the ($\bar{1}\bar{1}1$) bottom side facet on the opposite side of the nanoparticle, both oriented along the longer axis of the nanoparticle, exhibit a more positive strain state compatible with a 100% Pt surface concentration. The experimentally observed top surface 111 facet strain value is found to be 0.18%, pointing also to a 100% Pt surface composition.

We demonstrated that CXDI of a selected Pt_60_Rh_40_ alloy nanoparticle can be performed under realistic catalytic reaction conditions at elevated temperatures, retrieving the 3D nanoparticle shape and surface as well as bulk lattice strain state. Switching from reducing to active CO oxidation reaction conditions leads to a reduction of the surface strain state, which we resolve for individual <100> and <111> type facets. Through a rigorous comparison with DFT calculations, the facet strain state change can be attributed to gas environment–induced nanoparticle surface compositional changes from a pure Pt termination under reducing conditions to a Rh-rich termination under reaction conditions, varying for different facets. This process turns out to be nonreversible mainly for <111> type facets when switching back to reducing conditions. These facet-dependent, compositional heterogeneities may enable more effective reaction pathways and higher catalytic activity and selectivity. Our results open opportunities for surface-sensitive single-nanoparticle structural investigations of heterogeneous catalysts under industrial operando reaction conditions.

## MATERIALS AND METHODS

### Nanoparticle growth and pre/post-experimental characterization

PtRh nanoparticles were grown on a (001) oriented STO crystal. As previously described (*16*), a titanium oxide surface termination was induced following a protocol that includes etching in a buffered oxide solution and annealing in air (*34*). Pt and Rh were co-deposited by electron beam evaporation after heating the STO crystal to 1103 K in ultrahigh vacuum (UHV). Subsequently, the sample with the nanoparticles was annealed for 60 min under UHV at 1473 K. The nanoparticle composition was analyzed by energy-dispersive x-ray analysis and additionally determined from the d-spacing at 700 K using Bragg’s law and Vegard’s law (see the Supplementary Materials). To ensure the relocalization of nanoparticles preselected in the SEM (*35*), reference markers were deposited in close vicinity. Details on the marking, strategy of marker arrangement, and a relocalization protocol can be found in the Supplementary Materials and previous work (*16*). All SEM images were obtained at an acceleration voltage of 5 kV. Ex situ AFM topographic images were obtained in tapping mode in air using an oxide-sharpened silicon cantilever (*35*).

### Operando Bragg CXDI experiment

The CXDI experiment was performed at ESRF ID01 beamline, Grenoble, France (*36*). The beam energy was 9 keV, corresponding to an x-ray wavelength of 0.138 nm. Kirkpatrick-Baez mirrors were used to focus the beam to a size of ~400 nm by 400 nm at the sample position. The footprint size of the beam was about 1.3 μm along the beam for the used incident angle θ of 17.87° (see fig. S4 for a sketch of the experimental setup). The coherent x-ray flux was about 10^9^ photons per second. An Eiger2M detector with 1030 × 2164 pixels and a pixel size of 75 μm by 75 μm was mounted on the diffractometer arm at a sample to detector distance of 0.85 m. In situ CXDI was applied to track the structural dependence of a preselected nanoparticle on different gas environments. The 3D intensity distribution around the (111) Bragg reflection was collected by rocking the sample around the *y* axis (see fig. S4) within the Δθ range of ±1° from the angular position corresponding to the maximum of the scattered intensity. A total of 100 frames were acquired in the rocking scan, with an exposure time of 5 to 7 s per frame. During each step of the rocking scan, the signal was optimized by translational scans over 1 to 2 μm to compensate for the sample drift.

The gas environment was controlled by a custom-made gas mixing cabinet as described previously (*16*). Each gas flow was set by a calibrated mass flow controller. The reactor pressure was controlled by a backpressure controller, while the sample temperature was set by a calibrated boron nitride–covered resistive heater. The gas composition was determined by a mass spectrometer that was positioned between the outlet of the reaction chamber and the backpressure controller. A constant total flow of 50 ml/min and a pressure of 100 mbar were applied throughout the experiment. The sample was investigated under four gas environments as listed in table S1.

Before the CXDI experiment, the STO crystal–supported PtRh nanoparticle sample and the reaction chamber were cleaned using H_2_ (2 ml/min) and Ar (48 ml/min) at a temperature of 623 K for 25 min. During the experiment, a substrate temperature of 700 K was maintained throughout the whole reaction experiment.

### Phase retrieval

The 3D real-space complex object was reconstructed from the 3D reciprocal-space data using an iterative phase-retrieval approach. This complex object embeds information on the electron density distribution of the nanoparticle and on the projection of the strain field onto the wave vector transfer (*37*). For the phase retrieval, a combination of continuous hybrid input-output (100 iterations) and error reduction (300 iterations) including shrink wrap (with the threshold of 0.2) algorithms was used (*38*–*40*). This sequence was repeated twice, giving in total 800 iterations. Ten successful reconstructions were selected, each obtained from a different random initial phase, which were first aligned using a subpixel image registration algorithm (*41*) and then averaged to produce the final structure of the PtRh nanoparticle. The ranges in the reciprocal space data and real-space voxel size are reported in table S2. The modulus of the real-space object was normalized in the [0, 1] range, and all values less than 55% of maximum were set to zero.

Isosurface images (with 0.55 isosurface value) are shown in fig. S6, and slices corresponding to three cross sections are presented in fig. S8. The phase φ(***r***) of the real-space object is related to the displacement field ***u***(***r***) by the relation φ(***r***) = −***Q***·***u***(***r***), where ***Q*** is the scattering vector that corresponds to the crystallographic direction [111] in our experiment and is aligned along the *z* axis (see fig. S4). From the phase value, one can determine the *z* component of the displacement field *u_z_*(***r***) = φ(***r***)/|***Q***| and the strain field component ϵ*_zz_*(***r***) that is obtained by ϵ*_zz_*(***r***) = ∂*u_z_*(***r***)/∂*z* (*37*). Details on the determination of facet-dependent strain and the resolution are given in the Supplementary Materials.

### Computational details of the DFT investigation

All DFT calculations were performed using the projector augmented wave (PAW) method with the VASP program package in version 5.4.1, standard PAW potentials, and an energy cutoff of 400 eV (*42*, *43*). The PBE (*24*) functional was used with Grimme’s dispersion correction D3 (zero damping) (*44*). A Γ-centered *k*-point sampling using the Monkhorst-Pack scheme was used with *k*-point densities depending on the specific unit cells. For the cubic bulk unit cells of fcc Pt, Rh, or PtRh alloys (containing four metals atoms), a *k*-point sampling of 16 × 16 × 16 was used. For calculations on ($\sqrt{2}\times \sqrt{2}$)R45° and (2 × 2) unit cells for 100 and 111 surfaces, respectively, a *k*-point sampling of 6 × 6 × 1 has been proven to be sufficient. Last, for calculations that involve larger supercells (for example, where a RhO_2_ overlayer was included), a *k*-point sampling of 8 × 2 × 1 was used for (2 × 8) unit cells on the 100 facet and 1 × 1 × 1 for (8 × 8) unit cells on the 111 facet. Bulk fcc lattice constants were optimized with an increased energy cutoff of 800 eV. The lattice constant was subsequently kept fixed in surface calculations. Free energy corrections to the oxygen molecule were computed using the rigid-rotator harmonic-oscillator approximation at 700 K and a reference pressure of 2 mbar, giving a chemical potential of ∆μ_O_ = −0.92 eV relative to 0 K. The configuration of the structural models and the calculation of the Gibbs free energy of formation are described in the Supplementary Materials.

The mean strain was calculated in the following way: The strain is defined as the deviation of an interlayer spacing (111 or 100) with respect to the bulk value of this interlayer spacing. Because the experimentally determined strain was averaged over a voxel size of 2 nm along the scattering vector, a corresponding mean strain value was computed for comparison. This was achieved by averaging over an (integer) number of layers that comes closest to the voxel size of 2 nm, about 10 layers for 111 and 11 layers for 100. The averaged theoretical strain component $\epsilon_{\mathrm{zz}}^{t}$in the direction of the scattering vector was calculated using Eq. 1, where *h_i_* is the layer distance between layer *i* and layer *i* + 1 and ${\bar{h}}_{\text{bulk}}$ is the average layer distance at the bulk limit

$$\epsilon_{\mathrm{zz}}^{t}=\frac{\sum_{i=1}^{n}(h_{i}-{\bar{h}}_{\text{bulk}})}{n\times {\bar{h}}_{\text{bulk}}}\times 100\%$$ (1)

To be able to compare with the experimental ϵ*_zz_* values of the side facets, the theoretical $\epsilon_{\mathrm{zz}}^{t}$ values have to be transformed to

$$\epsilon_{\mathrm{zz}}^{t*}=\epsilon_{\mathrm{zz}}^{t}{\text{cos}}^{2}\alpha$$ (2)

where α is the angle between the facet and the substrate (α = 54° for <100> type side facets and α = 70°for <111> type side facets). The cos^2^α factor arises because of the geometrical projection on the side facet and the transformation of the gradient to the side facet surface normal direction.

${\bar{h}}_{\text{bulk}}$ was also determined from the slab calculations as the value to which *h_i_* converges in the central bulk region of the slab. Generally, the central ^1^/_3_ of the slab was used to determine ${\bar{h}}_{\text{bulk}}$, whereas the top and bottom ^1^/_3_ were treated as surface-like structures. Specifically, for nine-layer slabs, the interlayer spacings from *h*_3_ to *h*_5_ were used to determine ${\bar{h}}_{\text{bulk}}$, while for 11-layer slabs, the interlayer spacings from *h*_4_ to *h*_7_ were used to determine ${\bar{h}}_{\text{bulk}}$. Figure S13 depicts oscillations of layer distances *h_i_* from the surface layers to the bulk limit, in which different numbers of layers were used to calculate ${\bar{h}}_{\text{bulk}}$.

Reaching a slab size that is large enough to average over a relevant range of 2 nm was not feasible for some of the systems considered in this work. Instead, *h_i_* was calculated explicitly only for the top ^1^/_3_ of the slab that was considered as the surface region and the ^1^/_3_ that was considered the bulk region. This will generally provide on the order of 3 to 12 interlayer spacings *h_i_*. The remaining *h_i_* required to compute the strain over 2 nm were taken to be exactly ${\bar{h}}_{\text{bulk}}$. Note that including the actual *h_i_* of the bulk region that were used to determine ${\bar{h}}_{\text{bulk}}$ has the same effect as if one were to use ${\bar{h}}_{\text{bulk}}$ also for these layers.

## Supplementary Materials

### This PDF file includes:

Supplementary Text Legends for movies S1 to S4 Figs. S1 to S18 Tables S1 to S12 References
