# Supplementary material for: Single alloy nanoparticle x-ray imaging during a catalytic reaction
Source: Sci Adv. 2021 Oct 1;7(40):eabh0757. doi: 10.1126/sciadv.abh0757 (PMC10938497; doi:10.1126/sciadv.abh0757)
Supplement: Supplementary file 1 — Supplementary Text Legends for movies S1 to S4 Figs. S1 to S18 Tables S1 to S12 References [file sciadv.abh0757_sm.pdf]

Science Advances
AAAS

Supplementary Materials for

Single alloy nanoparticle x-ray imaging during a catalytic reaction

Young Yong Kim, Thomas F. Keller, Tiago J. Goncalves, Manuel Abuin, Henning Runge, Luca Gelisio, Jerome Carnis, Vedran Vonk, Philipp N. Plessow, Ivan A. Vartaniants, Andreas Stierle*

*Corresponding author. Email: andreas.stierle@desy.de

Published 1 October 2021, *Sci. Adv.* **7**, eabh0757 (2021)
DOI: 10.1126/sciadv.abh0757

The PDF file includes:

Supplementary Text
Legends for movies S1 to S4
Figs. S1 to S18
Tables S1 to S12
References

Other Supplementary Material for this manuscript includes the following:

Movies S1 to S4

Captions movies S1-S4

Movie S1.
Amplitude and strain distribution in 2D top view slices, from top to bottom, for Ar (I)

Movie S2.
Amplitude and strain distribution in 2D top view slices, from top to bottom, for Ar+CO (II)

Movie S3.
Amplitude and strain distribution in 2D top view slices, from top to bottom for Ar+CO+O_2 (III)

Movie S4.
Amplitude and strain distribution in 2D top view slices, from top to bottom for Ar+CO (IV)

Supplementary text:

Substrate preparation and nanoparticle growth and pre-/post experiment characterization

PtRh nanoparticles were grown on a (001) oriented $SrTiO_3$ (STO) substrate crystal with a (100) edge orientation (miscut < 0.1°, epi-polished). As previously described (*16*), the STO crystal was soaked in ultra-pure water for 10 min in an ultrasonic bath, etched for 30 sec in a buffered oxide etch solution (BOE, ammonium fluoride buffered hydrofluoric acid (6:1)), rinsed in high-purity water and dried in a stream of dry N_2. Afterwards, the STO crystal was annealed in a tube furnace in air at 1273 K (heating rate 1200 K/h) and kept at this temperature for 1 h to induce a titanium oxide surface termination (*34*).

After mounting the STO crystal on a Mo sample holder by spot-welding Ta clips onto the edges and the transfer into an ultra-high vacuum (UHV) chamber, the crystal was heated to 1103 K. Then, platinum (Pt) and rhodium (Rh) were co-deposited for 30 min by electron beam evaporation (target concentration 60 at% Pt, 40 at% Rh) from Pt and Rh rods. Subsequently, the sample was annealed for 60 min under UHV at a temperature of 1473 K to induce a nanoparticle size that is suitable for a CXDI experiment, a close to equilibrium shape (*45*), and to ensure a sufficient separation from adjacent NPs.

The nanoparticle composition was analyzed by energy dispersive x-ray analysis (EDX). The drift corrected EDX spectra in Fig. S3 were obtained at an acceleration voltage of 5.0 kV with a 150 mm^2 EDX detector (*35*). The chemical composition was determined by fitting the EDX spectra of the M_α platinum and the L_α rhodium peaks from the nanoparticle containing region in Fig. S3 using a filtered least squares approach (FLS, AZtec 3.0 software from Oxford Instruments). L_α strontium and K_α oxygen peaks in the EDX spectra from the neighborhood of the PtRh nanoparticles arise from the STO substrate. Similarly, a weak L_α Ti signal can be identified on the low energy shoulder of the K_α oxygen peak. The K_α carbon peak indicates that during the SEM imaging typically carbon is created on the sample surface. The average composition of the single

PtRh alloy particle was in addition determined from the d-spacing at 700 K using Bragg's law and Vegard's law. The distance of atomic planes in [111]-direction, d_{111}, is $d_{111} = \frac{a_{alloy}}{\sqrt{3}}$. The lattice constant of the alloy nanoparticle a_{alloy} can therefore be deduced from Bragg's law, yielding $a_{alloy} = \frac{\sqrt{3}\lambda}{2\sin\theta_{Bragg}}$, λ being the wavelength of the x-ray beam and θ_{Bragg} the position of the (111)-Bragg peak determined from the experiment. Following *Maisel et al.* and assuming that the binary PtRh alloy does not exhibit a miscibility gap (*21*), the PtRh composition can be estimated from Vegard's law, $a_{alloy} = x \cdot a_{Pt} + (1-x) \cdot a_{Rh}$. Here, x is the concentration of platinum in the alloy particle. The lattice constants at 700 K are a_{Pt}= 3.9387 Å and a_{Rh} = 3.8177 Å for Pt (*46*) and Rh (*47*), respectively. In the experiment, the Bragg peak position was determined to be θ_{Bragg} = 17.873° with an x-ray wavelength of λ = 1.378 Å, yielding a mean alloy composition of $Pt_{58.4}Rh_{41.6}$, close to the nominal composition and the results from the EDX analysis discussed below.

To ensure the re-localization of nanoparticles pre-selected in the SEM (*35*), reference markers were deposited in close vicinity by electron- and ion-beam-induced deposition (EBID/IBID) of a Pt precursor gas. In a three-level hierarchical way, additional guiding markers with increasing size and thickness were applied towards the edges of the STO single crystal substrate, e.g., see, a zero-order marker in Fig. S2, facilitating a simplified guided search across the PtRh nanoparticle landscape. Python and Matlab scripts were employed to translate the stage positions of the markers and nanoparticles in the SEM to the stage positions at the ID01 x-ray beamline and in the AFM. The Python script permits a direct import and export of the coordinate positions from the stage and the sample to the beamline operation system. PtRh nanoparticles were selected based on their size, shape and isolation to avoid parasitic scattering from adjacent particulates. Second order markers were placed within a distance of 50 μm from the selected PtRh nanoparticles to lie within the scanning range of the piezo stage at the x-ray beamline. The distortion of the x-ray scanning image in Fig. S2d as compared to the SEM image in Fig. S2c is due to the sample tilt to fulfill the Bragg condition. This implies that in specular geometry the piezo move parallel to the x-ray beam is accompanied by a change in sample height translating into an additional move along the x-ray beam, resulting in the apparent stretch of the x-ray scanning image. Further details on the marking, strategy of marker arrangement and a re-localization protocol can be found in a previous work (*16*).

All SEM images were obtained at an acceleration voltage of 5 kV. The lower resolution SEM images of the hierarchical guiding markers (Fig. S2) were taken with an Everhart-Thornley secondary electron (SE) detector. The higher magnification images of the nanoparticle in Fig. 1 were obtained with the through-lense detector (TLD) in the SE mode. Ex situ AFM topographic images were obtained in tapping mode in air using an oxide-sharpened silicon cantilever (*35*). The total size of the overview image in Fig. 1 was 30 µm × 30 µm with a resolution of 2048 lines and 2048 samples per line, and a scan rate of 0.41 Hz. For the size of the high-resolution AFM images of the single nanoparticle in Fig. 1, Fig. S1 and Fig. S7 a lateral scanning size of 550 nm × 550 nm and a scan rate of 0.44 Hz were chosen. The line scan in Fig. S1b was taken in the 2 µm× 2 µm topographic AFM image shown in Fig. S1a.

Bragg CDI data analysis

The 3D reciprocal space intensity distribution was obtained by linear interpolation of diffraction patterns on a Cartesian reciprocal space Q_x, Q_y, Q_z grid. Before interpolation the average background on the level of three photons was subtracted (from the Be dome, the surrounding gas or the substrate) and less than 0 counts were set to zero. Slices of the intensity distribution reported in Fig. S5 show clear interference fringes arising from the coherent diffraction of a faceted single particle. Small changes in the reciprocal space intensity distribution are noticeable, and are related to shape and strain field modification in the catalytic nanoparticle under different gas atmosphere conditions.

In practice, since the phase $\varphi(\boldsymbol{r})$ is reconstructed with an unknown offset, it was set to zero at the center of mass of the support obtained using a cutoff of 55%. Next, the phase was unwrapped and the displacement field $u_z(\mathbf{r})$ was calculated (see Fig. S6 and Fig. S8). To determine the strain, the derivative of the displacement field was taken as it is shown in Fig. S8 and Fig. S9.

The real space resolution was determined by two different methods. The first one was the Phase Retrieval Transfer Function (PRTF) (*48*), with resolution values determined at a threshold 1/e. The second approach was based on the procedure proposed in (*49*). In this method, the resolution is defined as the FWHM of the point spread function (PSF) that was obtained, in our case, by the blind deconvolution algorithm (*50,51*) which was implemented in MATLAB software. Importantly, the number of iterations was small and was determined as a first minimum of the amplitude mean-square error function. That gave us from 10 to 15 iterations in each gas conditions

case. This deconvolution process was applied to the amplitude function (threshold 0.55). The PSF from the amplitude of the object represents the degree of blurring for the particle along each direction. To determine the FWHM of the PSF in different directions they were fitted by Gaussian functions (see Fig. S12 and Table S3).

Determination of facet dependent strain

The outer layer of each facet of the reconstructed nanocrystal was isolated in order to extract facet-resolved strain histograms and compare them for different gas environments (Fig. S11). The data segmentation follows the workflow described in (*52*) with adaptations to the particular problem of performing statistics on the surface strain. It takes as input the reconstructed modulus and strain obtained by phase retrieval, as well as the isosurface value defining the surface voxel layer of the nanocrystal. The analysis can be divided into two parts: first, finding the number of independent facets, labelling them and getting a first estimate of the equations of planes parallel to them. The second step consists in refining the plane parameters by matching it with the reconstructed nanocrystals surface and isolating the voxels belonging to each facet.

In order to identify and label facets, the modulus is first meshed using Lewiner marching cubes (*53*), and then smoothed using Taubin's smoothing (*54*). Each triangle of the mesh is described by its vertices and normal. The normals are weighted by the surface of their respective triangle. Then, a density map of the same size as the array of normals can be created by summing for each normal the neighboring normals weighted by their distance, if they are closer than a certain radius. The next step is to project this 4D data (three positions and the density) using a stereographic projection (*55*), which provides two 2D plots corresponding to the projections from the South Pole and North Pole, respectively. Densities are then inverted, and new maps corresponding to the distance of the data to the background are calculated. From these distance maps, the local minima are identified, and labels are assigned to them. Then, watershed segmentation is applied in order to assign a label to each point of the projections (label 0 being the background). The duplicity of labels (two labels for a single facet) is checked using the position of the corresponding points on the stereographic projection. Now that the facets have been identified uniquely and labelled on the stereographic projections, one can go back to the corresponding normals, mesh vertices and finally voxels using array indices which are preserved during all calculations. Note that these voxels may not anymore

exactly correspond to the original object due to smoothing. They are used as an initial population of voxels belonging to a particular facet (label) in order to estimate the equation of a parallel plane. The first estimate of the plane equation is determined by minimizing the distance of the label's voxels to it. Then, the plane is translated along its normal in order to match it with the surface voxel layer of the nanocrystal, as defined by the isosurface value. The list of surface voxels belonging to the facet is updated using their distance to the plane, and the plane equation further refined. Finally, the crystal edges are isolated using a threshold on their coordination number, and the corresponding voxels are excluded from the list of voxels belonging to the facets (labels). The analysis script for the determination of the facet dependent strain is available on public repositories (*56*). A similar procedure to determine facet orientation dependent strain was also used in *(57)*.

Computational Details of the DFT investigation

Structural models

The bulk structures of PtRh alloys have already been studied in the literature and it has been found that – at T = 0 K – the most stable structure for a 50:50 PtRh alloy is the so-called "40" structure. However, it has also been found that these ordered bulk alloy structures already become unstable at temperatures below 300 K, with random alloys becoming more stable in free energy (*21*).

Our computational study does therefore not aim at determining the energetically most stable bulk composition for certain experimental conditions and compositions. Instead, we start with a fixed bulk composition and investigate the possible surface segregation in the 1st and 2nd surface layer as a function of external conditions. As the bulk structure, we focus on $L1_0$ $Pt_{0.5}Rh_{0.5}$, because it is similar to the experimental composition ($Pt_{60}Rh_{40}$) and because it is a simple structure that allows the construction of surface and interface models with relatively small unit cells. The surfaces fcc(100) and fcc(111) were constructed using the optimized bulk alloy to the lattice constant of 3.85 Å as slab models, which were separated by at least 15.5 Å of vacuum.

Calculations of the stability

The energetic stability of surface models, expressed by G_{form}, the Gibbs free energy of formation, is always computed with respect to a stoichiometrically terminated slab with bulk composition (typically $Pt_{50}Rh_{50}$):

$$G_{form} = E_{slab} - E_{slab,ref}^{stoich} - n_O/2(E_{O2} + \Delta\mu_{O2}) + \Delta n_{Pt}(\mu_{Rh} - \mu_{Pt}). \qquad \text{(S1)}$$

Here, E_{slab} is the total energy of the slab under consideration, $E_{slab,ref}^{stoich}$ is the total energy of the stoichiometric reference slab. n_O is the number of oxygen atoms, E_{O2} is the energy of the oxygen molecule and $\Delta\mu_{O2}$ is the chemical potential of the oxygen molecule at 700 K and 2 mbar reference pressure relative to the value at 0 K, which is $\Delta\mu_{O2}$ = -0.92 eV. Δn_{Pt} is the number of Rh atoms that are exchanged by Pt with respective to the reference structure (Δn_{Pt} can also be negative) and μ_{Rh} and μ_{Pt} are the total energies per atom of Rh and Pt, respectively, in their pure fcc bulk structures.

Clean and oxygen-covered 100- and 111-alloy surfaces

General trends: Platinum tends to migrate to the 1st surface layer for clean surfaces. Oxygen adsorption is typically exergonic, favors rhodium migration to the 1st layer and leads to an increase in strain. While the stability does not change significantly with the composition of the second layer (shifts in stability up to 0.1 eV, where low Pt content is preferred), the strain increases with the amount of Pt on the second layer. Interestingly, oxygen adsorbs most strongly on the bridge-position of the 100-surface also with 100% Rh in the first layer, which is not expected since the four-fold coordinated position is more stable on Rh(100). This is due to the different sub-surface composition in the 2nd and lower layers as well as the lattice constant of the alloy which is larger than that of Rh. An overview of the data is shown in Tables S4-S7.

A second layer composition of 100% Rh (75%) for the (100) ((111)) surface is in many cases energetically degenerate to the stoichiometric case, but the calculated average strain disagrees with the experimental values, ruling out this configuration (see Tables S4 and S5).

Effect of the slab thickness in relative stabilities and strain

The relative stability of PtRh alloys was calculated using equation S1, where 100- or 111-clean surfaces with a Pt/Rh ratio of 1:1 were used as references. Table S6 shows the mean strain averaged over 2 nm thickness and relative stabilities of PtRh alloys with different number of layers. While relative stabilities appear to be unaffected by the number of layers used (up to a difference of 0.02 eV/surface atom), the mean strain is more sensitive. 9-layer slabs deviate up to 0.07% when

compared to 18-layer slabs, while 11-layer slabs appear to be closer to convergence without a significant impact in computational performance, deviating only up to 0.04%. For this reason, strain and relative stabilities were computed using 11-layer slabs for $(\sqrt{2} \times \sqrt{2})$R45° and (2×2) unit cells.

For structures in which a RhO_2 overlayer was also included (Tables S8 and S9), 2×8 and 8×8 unit cells were considered for the 100 and 111 surfaces respectively to compensate for the lattice mismatch (different lattice constants between the PtRh alloy and the RhO_2 overlayer). Due to computational limitations, only 9-layer and 6-layer slabs were used for such structures.

Accuracy of Density Functionals for the prediction of relative stabilities and strain

In order to test to which extent the results depend on the choice of functional, we have additionally performed calculations with the BEEF-vdW functional. The relative stabilities of PtRh alloys for clean surfaces using PBE-D3 and BEEF-vdW functionals agree quite well, having deviations up to 0.02 eV/surface atom. Neglecting Van der Waals interactions (PBE functional) leads to deviations on the order of 0.1 eV/surface atom compared to PBE-D3 or BEEF-vdW. For oxygen-covered surfaces, deviations between PBE-D3 and BEEF-vdW are, however, as high as 0.04 eV/surface atom per oxygen adsorbed, leading to error accumulation for highly-covered surfaces. For instance, the adsorption of three oxygen atoms along with Rh segregation has a deviation of around 0.12 eV/surface atom between PBE-D3 and BEEF-vdW. However, predictions made regarding the most stable structure and changes in computed mean strain are largely similar. This corroborates the use of PBE-D3 and indicates that the result regarding mean strain and stability do not depend strongly on the functional, at least for the functionals studied herein.

While the calculated average strain values of different slabs depend on the exchange and correlation functional used for the structure relaxation, deviations appear to be systematic. According to Table S7, the mean strain of structures relaxed with the PBE-D3 functional are 0.05-0.12% higher than those relaxed with the BEEF-vdW functional, however the mean strain referenced to a clean slab with a layer composition of 50% Pt and 50% Rh does not exceed a difference of 0.02% between different functionals. This means that changes in the mean strain due to segregation or molecular adsorption are predicted more similarly with different functionals.

RhO$_2$-thin oxide films on the 100- and 111-alloy surfaces

Models of 100 and 111 PtRh surfaces with an RhO$_2$ overlayer are shown in Figs. S14 and S15 and were constructed as in previous work on RhO$_2$ overlayers on Rh and RhPt alloys (*31, 58*). Specifically, for 111, we use a $7 \times 7/8 \times 8$ supercell. For 100, we use a $\sqrt{2} \times 7/2 \times 8$ supercell (*57*). The energy difference between different terminations in the first metallic layer below the RhO$_2$ layer is much lower than energy difference for clean surfaces (Tables S8 and S9). This can be explained by the fact that the less stable alloy terminations are usually more reactive towards the RhO$_2$ overlayer, which partially compensates the different stabilities of clean surfaces. Consequently, no clear conclusion is reached, based on calculations alone, regarding the composition of the interface.

When the RhO$_2$-overlayer is included in the calculation of the mean strain, high values of >3% are obtained. This is because the distance of the Rh-atoms in the RhO$_2$-layer with respect to the metal atoms in the next metal layer is on the order of 3 Å, which is an increase of around 50% with respect to the metallic bulk spacing that is around 1.9 Å for 100 and 2.2 Å for 111.

When the RhO$_2$-overlayer is not included in the calculation of the mean strain, mean strain values are obtained, which are on the same order as those of clean surfaces. The dependence of the strain on the composition, however, is similar to clean or oxygen surfaces, e.g. a higher Rh content leads to lower strain and higher Pt content to higher strain.

Adsorption of CO on 100- and 111-surfaces

Adsorption energies were computed on the 100- and 111-surfaces using $(\sqrt{2} \times \sqrt{2})$R45° and (2×2) unit cells respectively with various terminations. Adsorption free energies were computed based on a rigid-rotator harmonic oscillator and free translator treatment of CO as well as a harmonic oscillator treatment of adsorbed CO on the pure Pt(111) surface. The corresponding free energy correction was applied to all surfaces. Corrections to the adsorption energies were derived from experimental values for Rh(111) and Pt(111) taken from (*29*) and Pt(100) taken from (*59*). The adsorption energies derived from experiment for CO are ΔE = -1.47 eV on Rh(111), -1.29 eV on Pt(111) and -1.51 eV on Pt(100) (*29*). These values exclude the zero-point vibrational energy (ZPVE) correction which was removed from the original experimental values based on computed ZPVE (0.08 eV for Rh(111), 0.08 eV for Pt(111) and 0.07 eV for Pt(100)). This leads to a direct comparison between experimental and calculated energies computed with PBE-D3 functional (-

2.03 eV, -1.91 eV and -2.31 eV respectively). In order to account for the systematic DFT error, we add a correction $\Delta E_{corr.}^{exp.}$ to the computed ΔE for CO adsorption, which is 0.59 eV for 111 surfaces (averaged over Rh(111) and Pt(111)) and 0.76 eV for 100 surfaces (derived from Pt(100)). $\Delta E_{corr.}^{exp.}$ for the 100-surface includes the systematic error of the theoretical value of Pt(100), which is 0.80 eV, plus an increment that corresponds to the difference between the error of the theoretical value averaged over Rh(111) and Pt(111) and the error of Pt(111), which is -0.04 eV.

The adsorption free energy for a given surface was thus obtained from the adsorption energy ΔE_{ads} computed for that surface, by adding the correction for adsorption energy derived from experiments plus the correction from thermal motion of the nuclei:

$$\Delta G_{ads} = \Delta E_{ads} + \Delta E_{corr.}^{exp.} + \Delta G_{harm}. \quad \text{(S2)}$$

The results are compiled in Table S10 and S11 and the most stable structures for the 111-surfaces are additionally shown in Fig. S16. Figure S17 shows the atomic structure of the most stable computed 111 surface with a quarter monolayer of CO. We find that, at the experimental conditions, surfaces are predicted to be clean, Pt-terminated surfaces. The most stable structure containing adsorbed CO in a 111-surface, is only 0.04 eV less stable. In this structure, a single Rh atom per 2×2 cell is in the first layer and one CO molecule is adsorbed in the on-top position on this Rh atom.

PtRh Nanoparticle/STO interface energetics

The computational model (shown in Fig. S18) is constructed using the Ti-terminated STO(100). The supercell is $2\times7\sqrt{2}$ -Pt(111) on $\sqrt{2}\times6\sqrt{2}$-STO(100). According to Table S12, the mean strain values are similar to those obtained for clean PtRh slabs without the STO support (Table S5). We also observed that relative stabilities per surface atom of 50% Pt / 50% Rh, pure Pt or pure Rh at the interface are very similar, so that there is no clearly preferred termination. The orientation of the metal-support interface is illustrated in Fig. S18.

Surface Energy calculations

Surface energies were determined from a series of slab calculations with increasing thickness (*58*) to obtain the bulk limit by extrapolation. For Pt-terminated 111 and 100 surfaces, we obtain 145.6

and 170.1 meV/Å^2, respectively. These values differ by only 1 meV/Å^2 from those of Pt and the unit cell of the alloy, which means that the difference to clean Pt surfaces is only due to the lattice constant of the alloy. The ratio of the surface energies 111/100 is 0.86. Adhesion energies were computed for the interfaces described above. If these adhesion energies are referenced to the most stable clean metallic surfaces (Pt-terminated) then we obtained –105.6 meV/Å^2 for the Pt-terminated interface and -108.1 meV/Å^2 for the stoichiometrically terminated interface.

Figures S1-S18

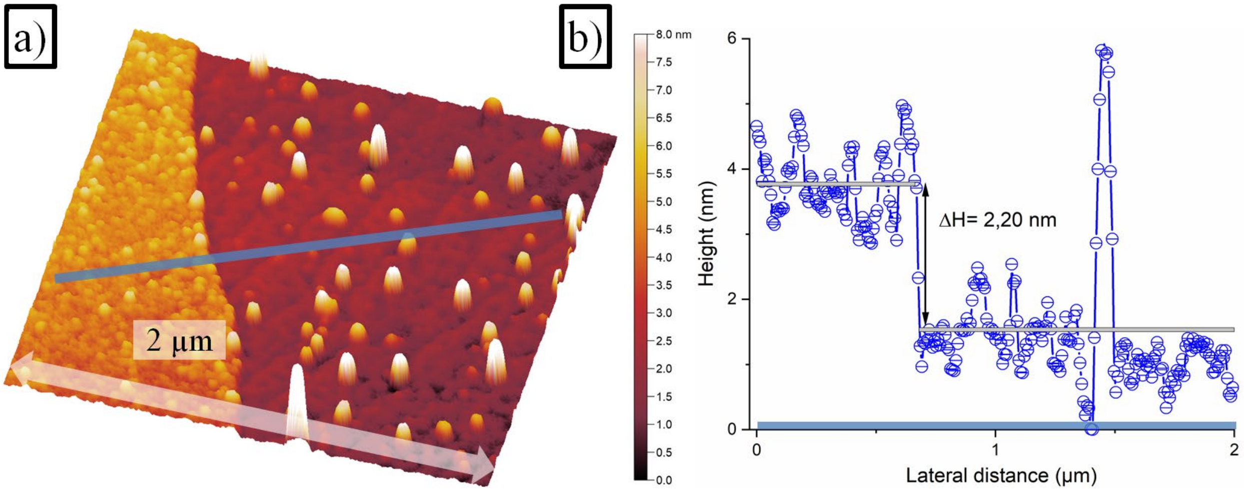

Fig. S1

Nanoparticle ensemble morphology characterization by AFM. a) 2D topographic AFM image showing the region of interest. A local variation in surface termination of the single crystal STO substrate induced a modification of the surface wetting properties and in turn results in a different nanoparticle growth behavior. The lateral image size is 2 μm × 2 μm, b) the line scan across the blue line indicated in a) indicates a step height of around 2.2 nm, whereas the nanoparticle height can be significantly higher depending on the region.

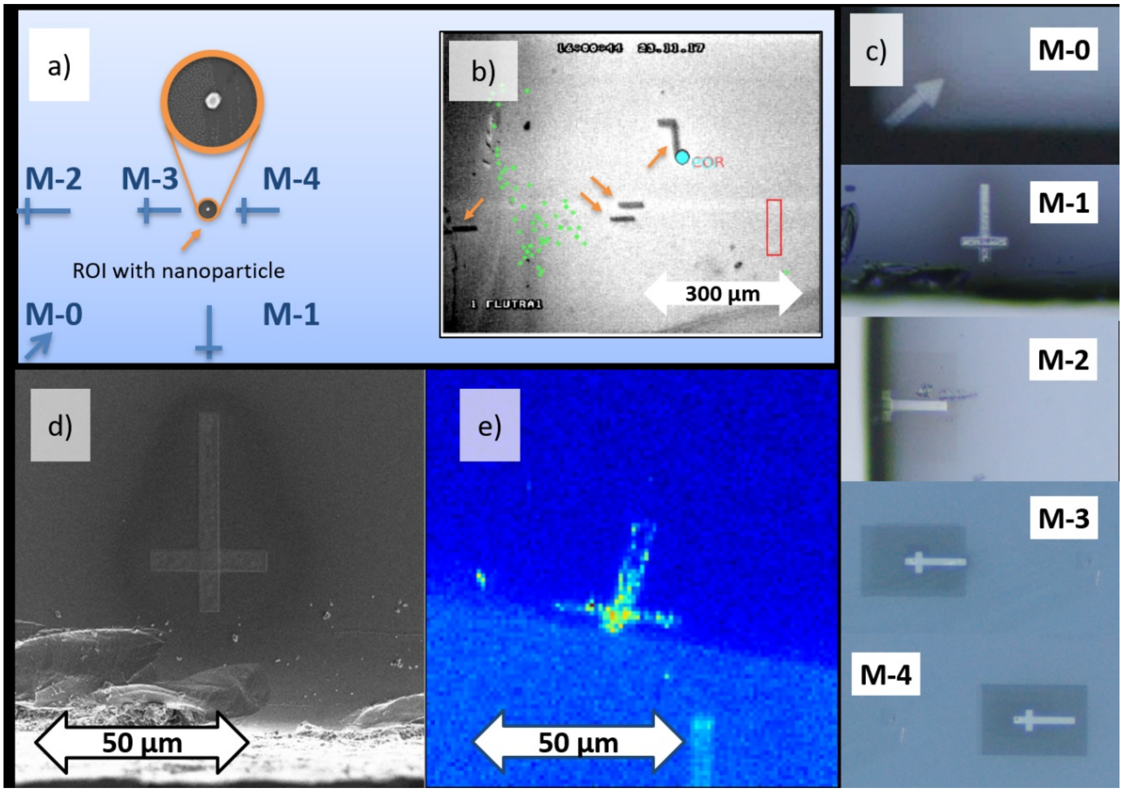

Fig. S2

Hierarchical markers for nanoparticle relocation. a) Scheme of the hierarchical marker arrangement on the STO crystal (sketched in blue). The markers are positioned in the lower left part of the 10 mm × 5 mm sized substrate to locate the nanoparticle in the region of interest (ROI), b) Snapshot image of an optical micrograph taken from the optical microscope mounted on the beamline. Several markers are visible and indicated by the arrows. c) optical microscopy images of the zero (M-0), first (M-1, M-2) and second (M-3, M-4) hierarchy level Pt markers at the sample edge, sample corners and in close vicinity to the nanoparticle containing region of interest, d) SEM image of marker M-1 and e) x-ray scanning image of the specular Pt 111 intensity distribution using the fast continuous scan of the piezo stage with the mounted crystal around marker M-1. The scanning range is 100 µm × 100 µm. The arrow appears distorted due to a misalignment between the sample surface plane and the piezo x-y scanner.

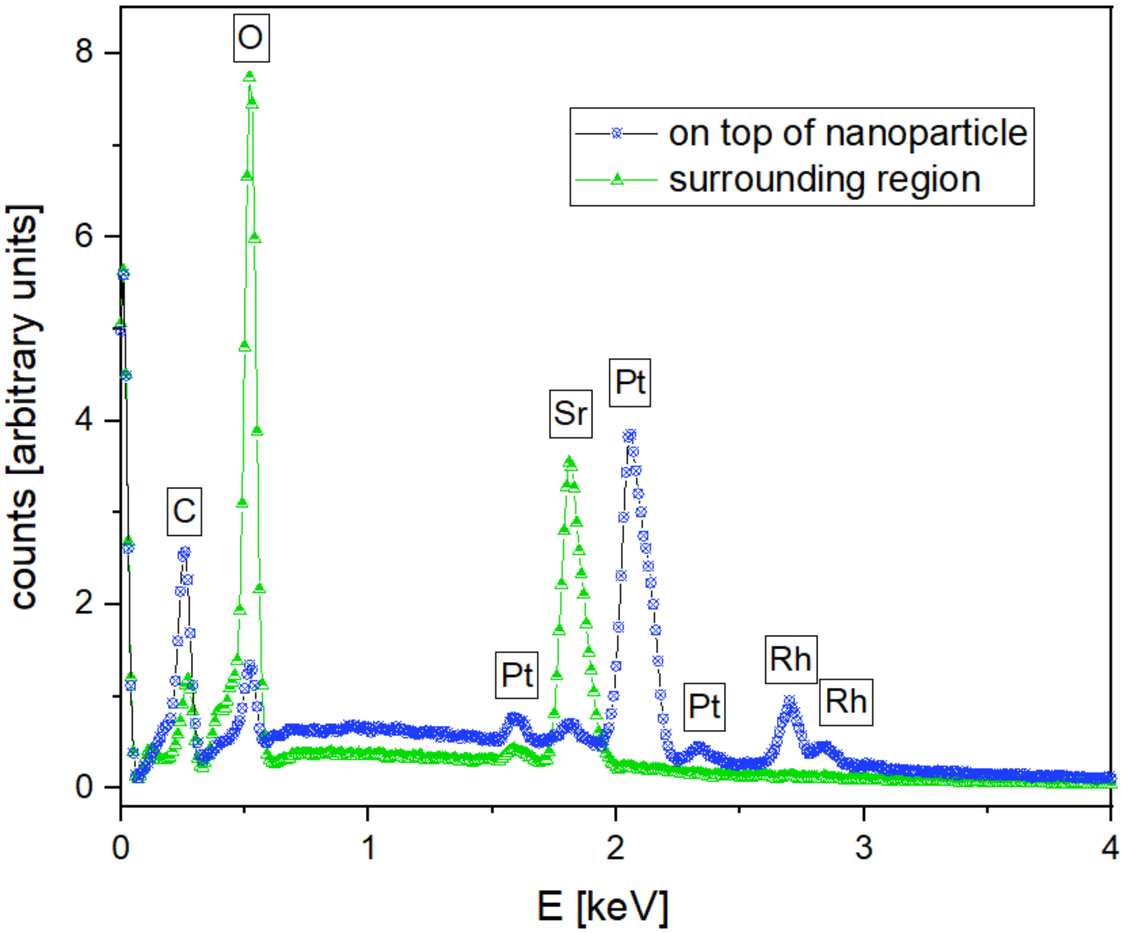

Fig. S3

EDX spectra from a PtRh nanoparticle (blue crossed circle) and from the less nanoparticle covered surrounding region (green open triangle).

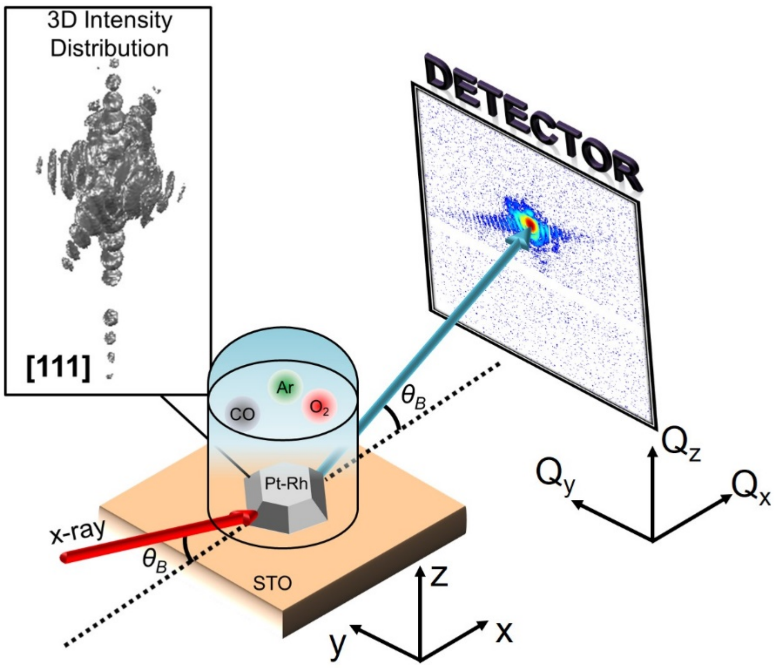

Fig. S4

Sketch of the setup of the *operando* CXDI experiment at the beamline ID01 at ESRF. The incident focused x-ray beam (shown in red) is interacting with the pre-selected PtRh nanoparticle inside the Be dome at Bragg θ_B =17.87° corresponding to the Pt 111 reflection. The outgoing x-ray beam (shown in light blue) is propagating to the detector, where the 2D diffraction patterns are recorded. To obtain the 3D scattered intensity, rocking scans in the angular range $\Delta\theta = \pm 1°$ were acquired. The geometrical directions are defined as indicated in the figure, z corresponds to the 111 direction. Inside the Be dome, Ar, CO, and O_2 atmospheres are established by a computer-controlled gas mixing cabinet with mass flow controllers.

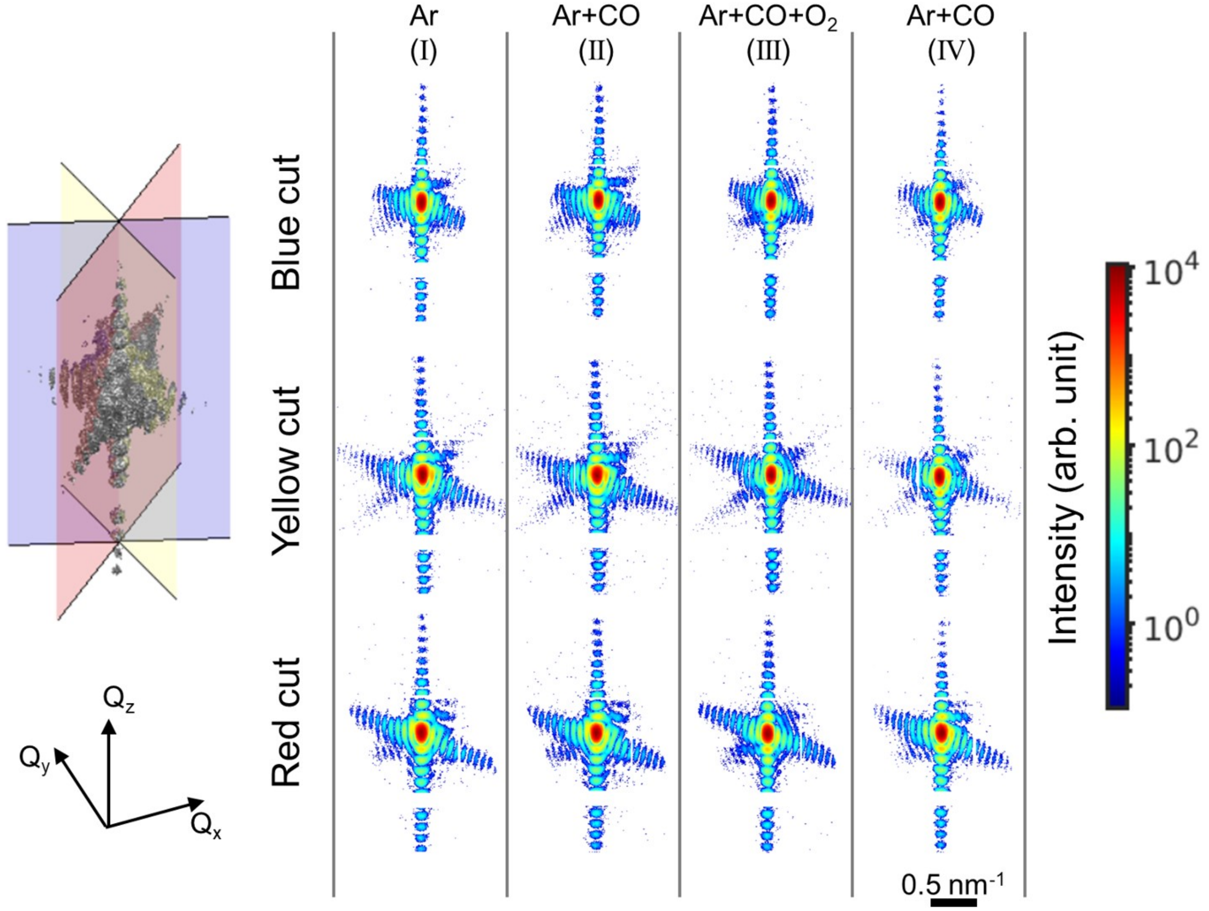

Fig. S5

Reciprocal space cuts through the 3D intensity distribution from the PtRh nanoparticle in three different directions shown in the inset.

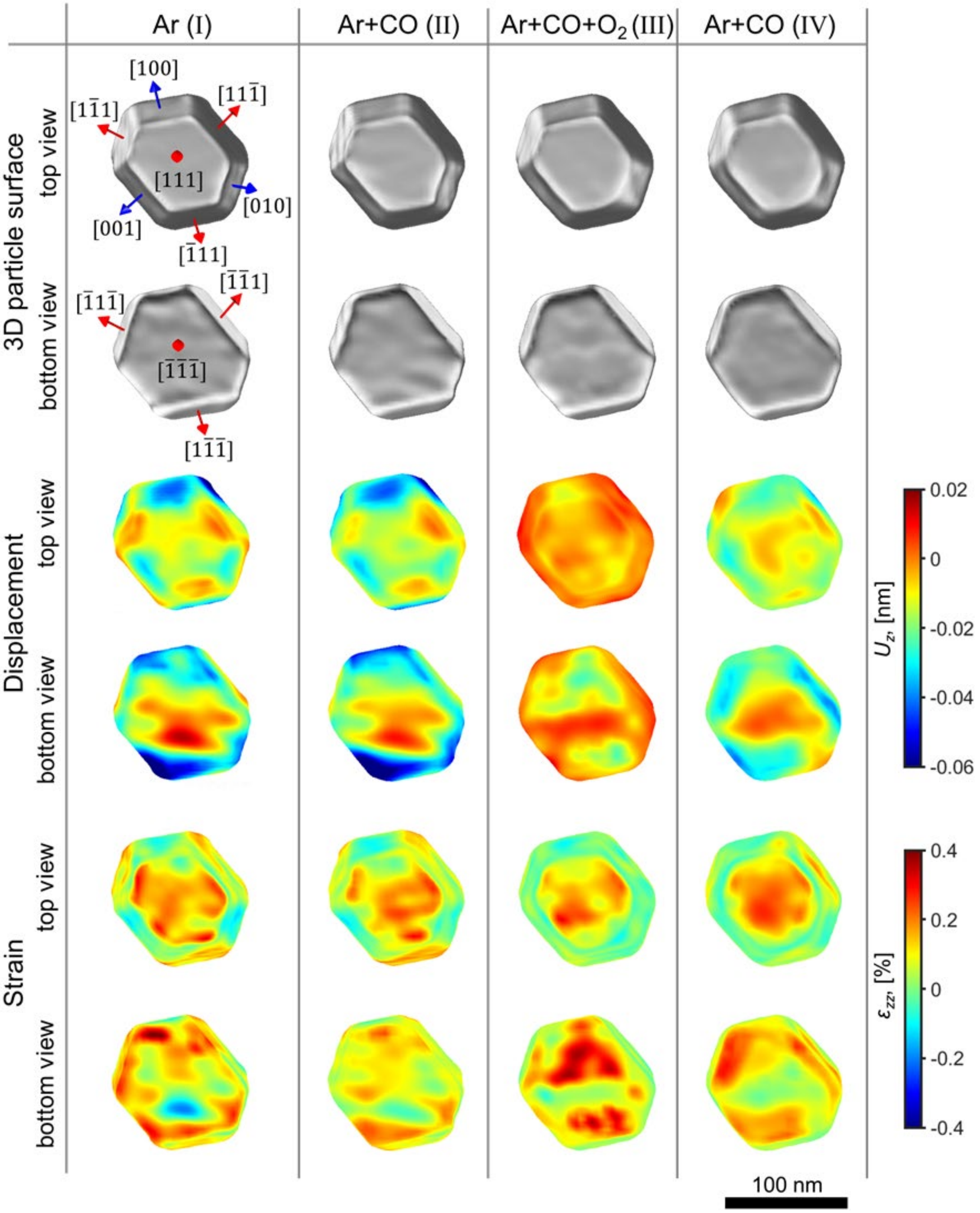

Fig. S6

Dependence of the PtRh nanoparticle surface structure on the gas environment. For each of the 3D particle surface, displacement, and strain, the first and second row show the top and bottom view of the 3D reconstructed PtRh nanoparticle, respectively.

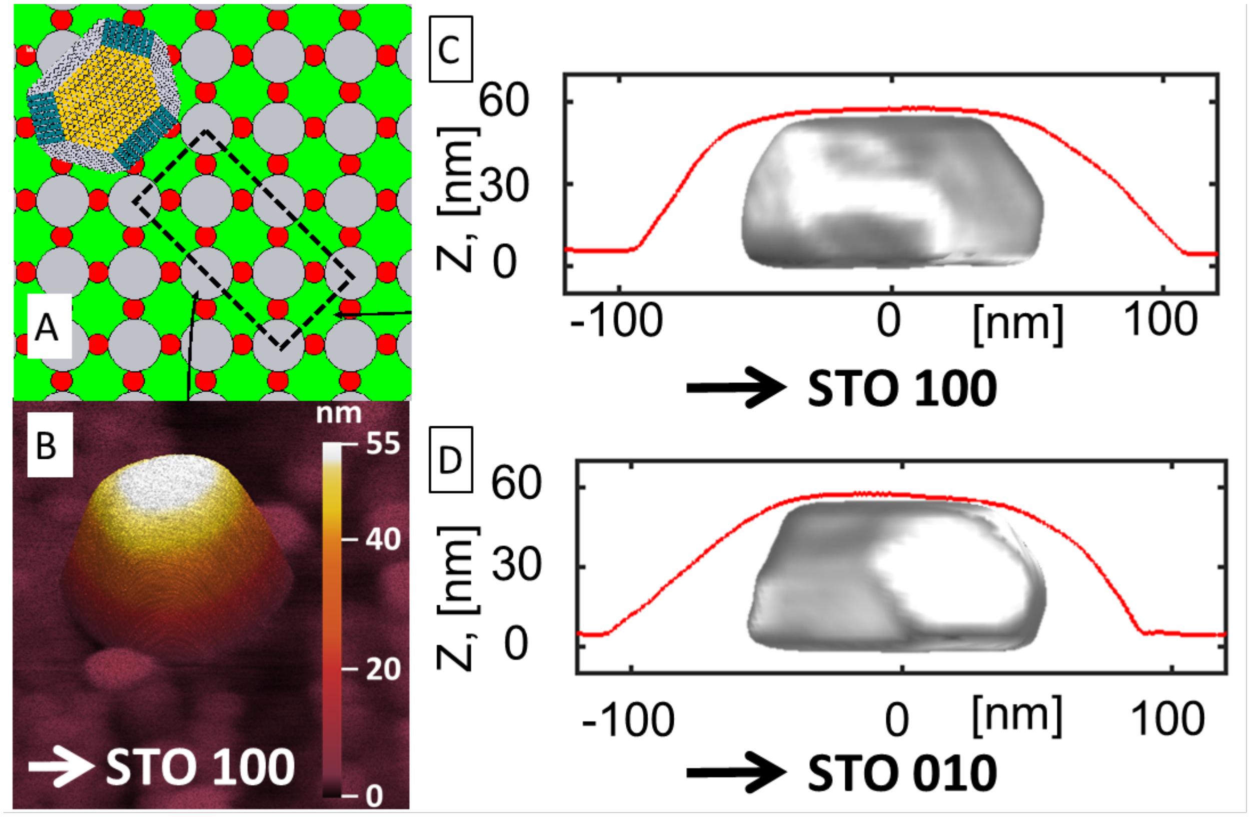

Fig. S7

Morphology of the investigated PtRh nanoparticle. (A) Schematic model of the relationship between PtRh nanoparticle and STO substrate. The rectangular box corresponds to the unit cell used for the DFT calculations. (B) AFM image and 100 direction of STO substrate direction. (C, D) To scale overlay of AFM profiles along STO 100 and STO 010 direction (red lines) and the corresponding orientation of CXDI reconstructions. The AFM height profiles appear wider than the CDI reconstruction due to the tip broadening effect originating from the convolution of the width of the nanoparticle and the AFM tip.

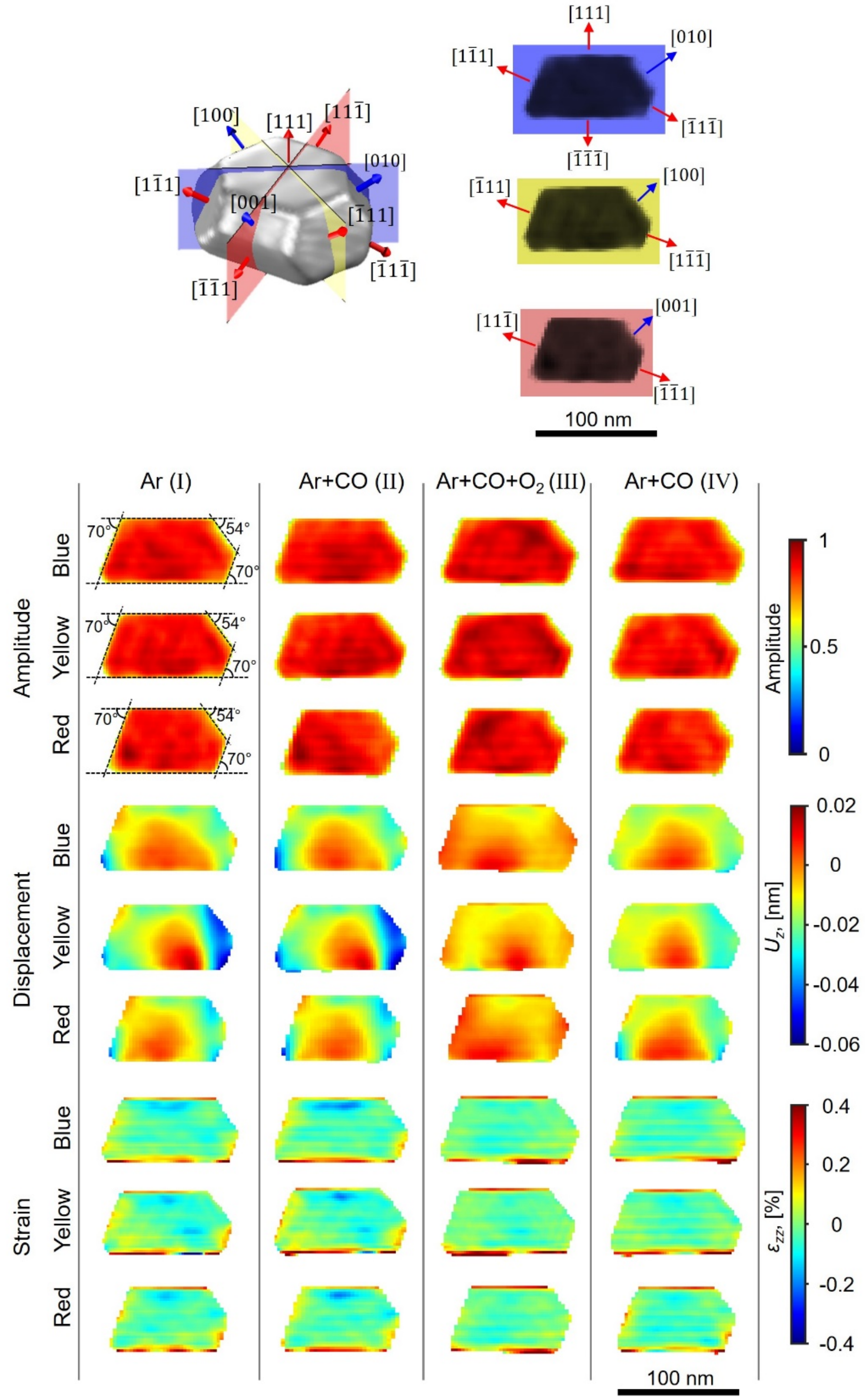

Fig. S8

Cross sections through the reconstructed PtRh nanoparticle for different gas conditions as defined in the top inset. Each column displays the reconstructed amplitude, displacement, and strain for a particular gas environment.

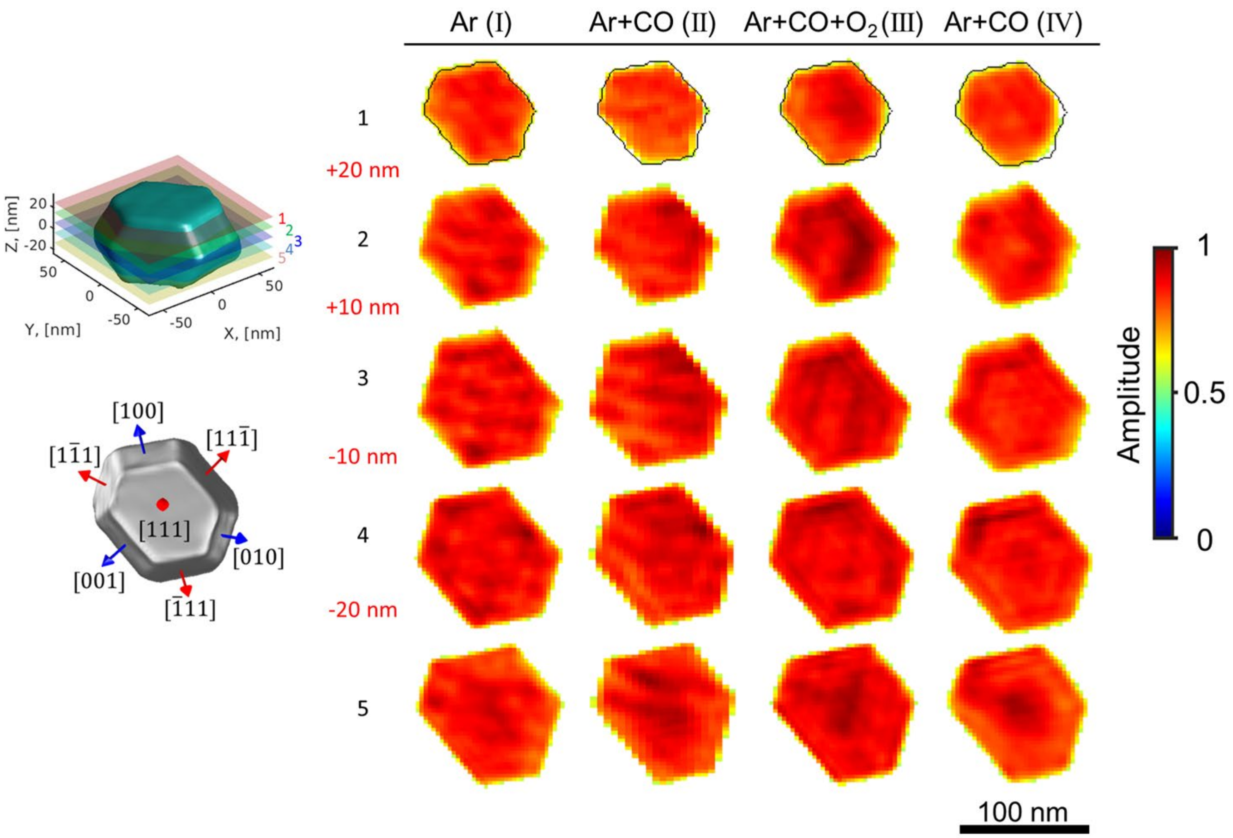

Fig. S9

Amplitude distribution in the reconstructed PtRh nanoparticle as a function of the gas environment shown for slices at different height levels as defined in the left inset. For the top slice of +20 nm we show the contour plot that is drawn around the initial case of the particle at Ar (I) conditions. The same contour plot is drawn for other gas conditions. The rounding of the top part of the particle is well seen in the case of Ar+CO (IV) gas conditions.

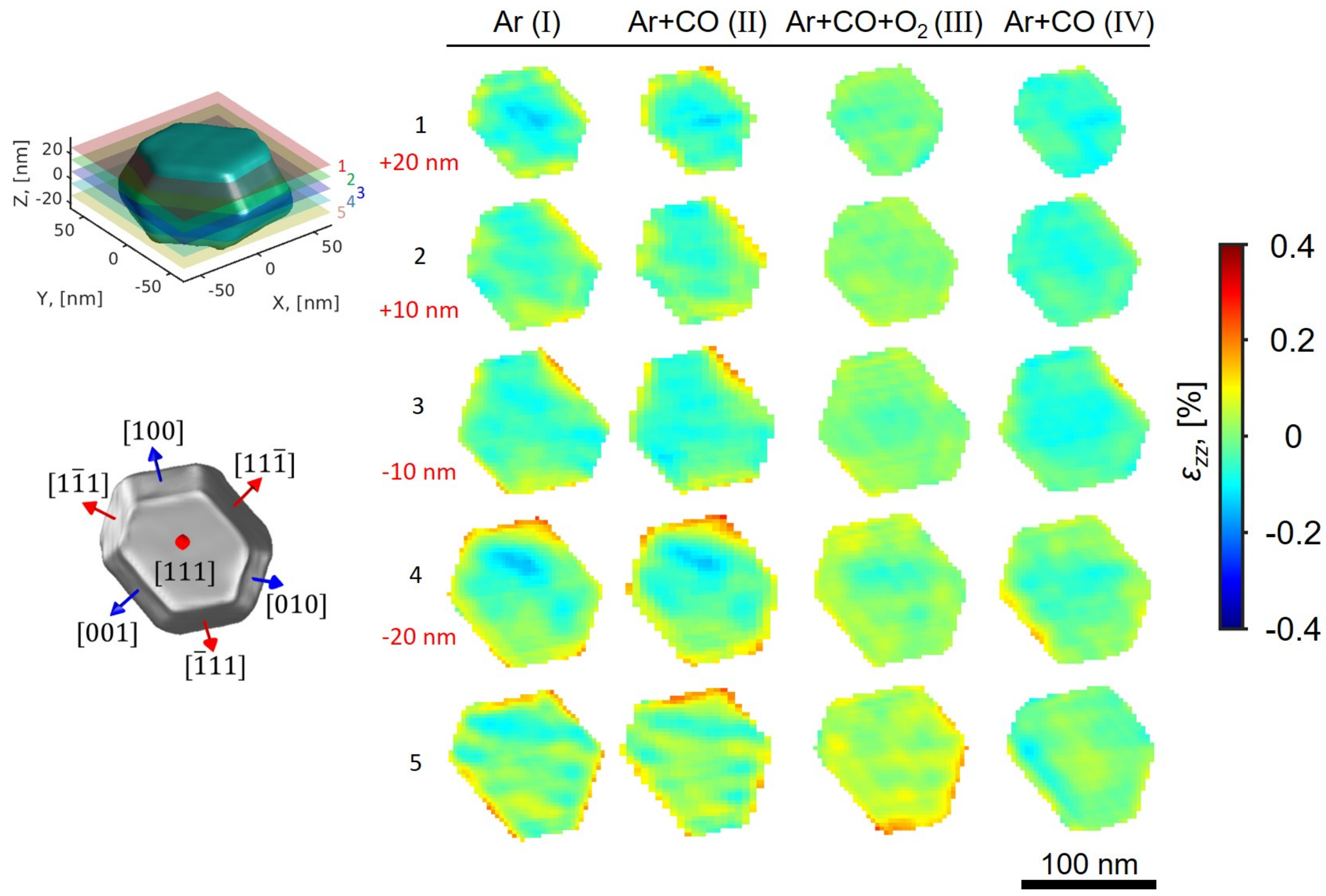

Fig. S10

Strain distribution in the reconstructed PtRh nanoparticle as a function of the gas environment shown for slices at different height levels as defined in the left inset.

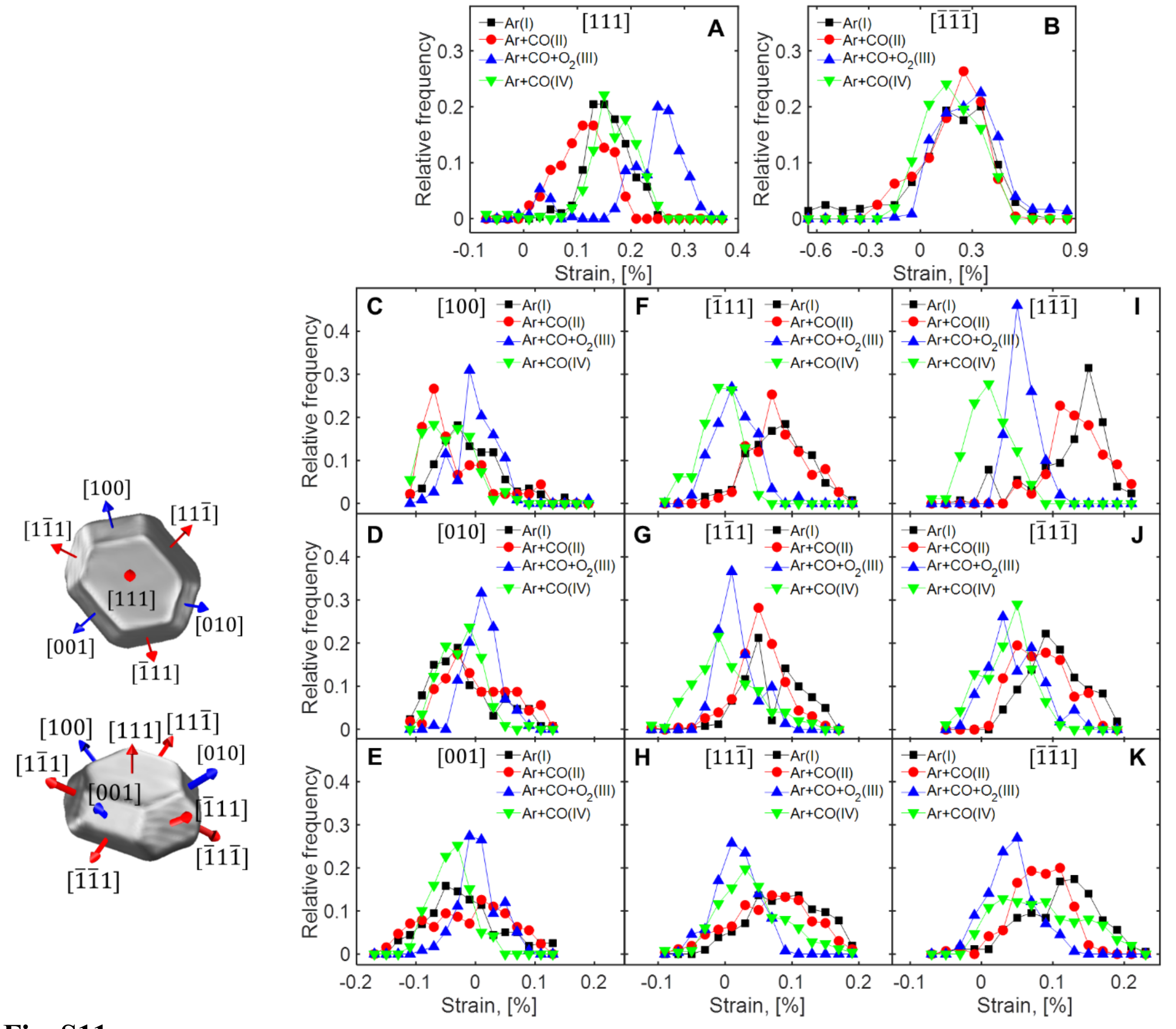

Fig. S11

Histogram of the strain for each facet. (A,B) upper and lower 111 facets, (C-E) 100 facet family, (F-H) upper and (I-K) lower 111 facet family.

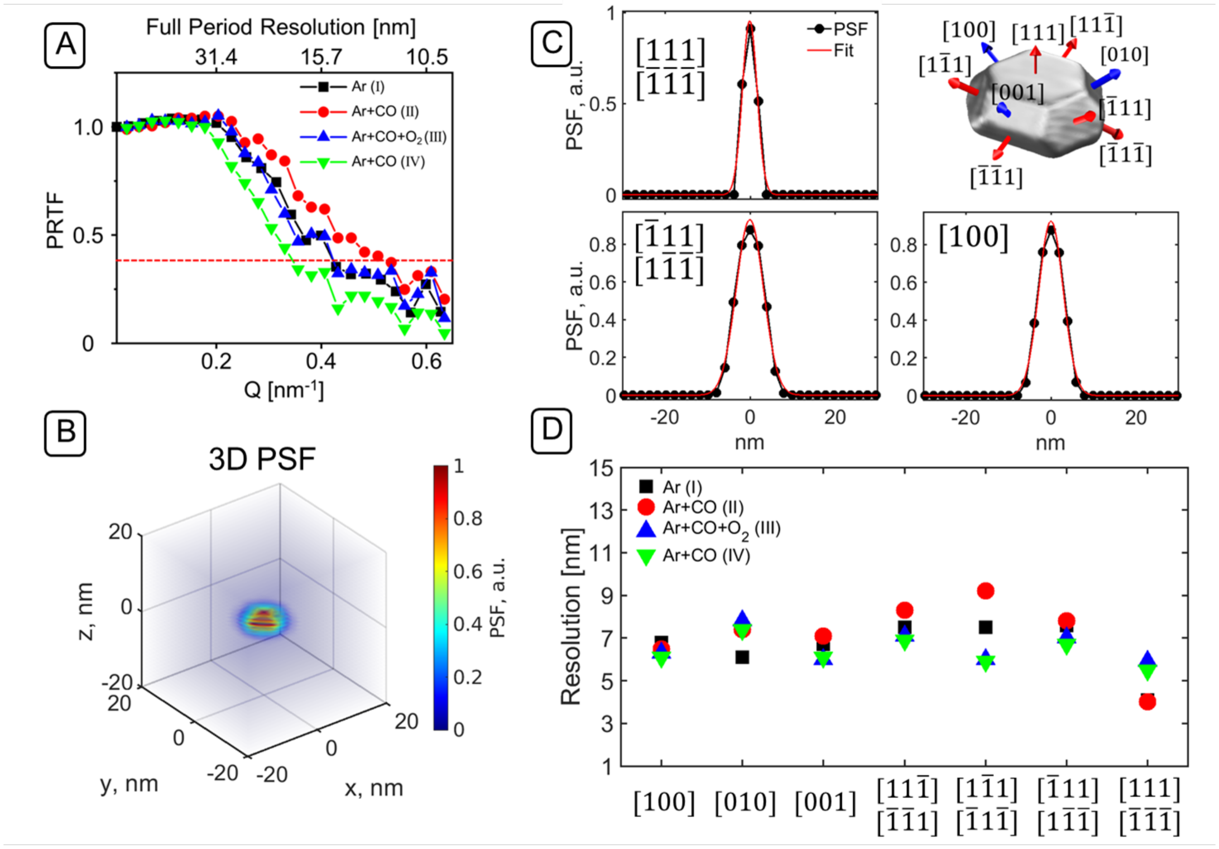

Fig. S12

Estimation of the CDI experimental resolution. (A) phase retrieval transfer function (PRTF) and estimated resolution under Ar (I), Ar+CO (II), Ar+CO+O2 (III), and Ar+CO (IV) gas atmosphere. The red dashed line at the 1/e level is used to define the resolution obtained in the reconstruction. (B) Three-dimensional Point Spread Function (PSF) obtained from the deconvolution process for the case of Ar (I). (C) Section of the PSF shown in (B) corresponding to selected nanoparticle facets. The PSF sections are represented with black circles, the red lines are results of Gaussian fits. (D) Resolution (FWHM of the Gaussian fit) plot for different facets under various gas conditions.

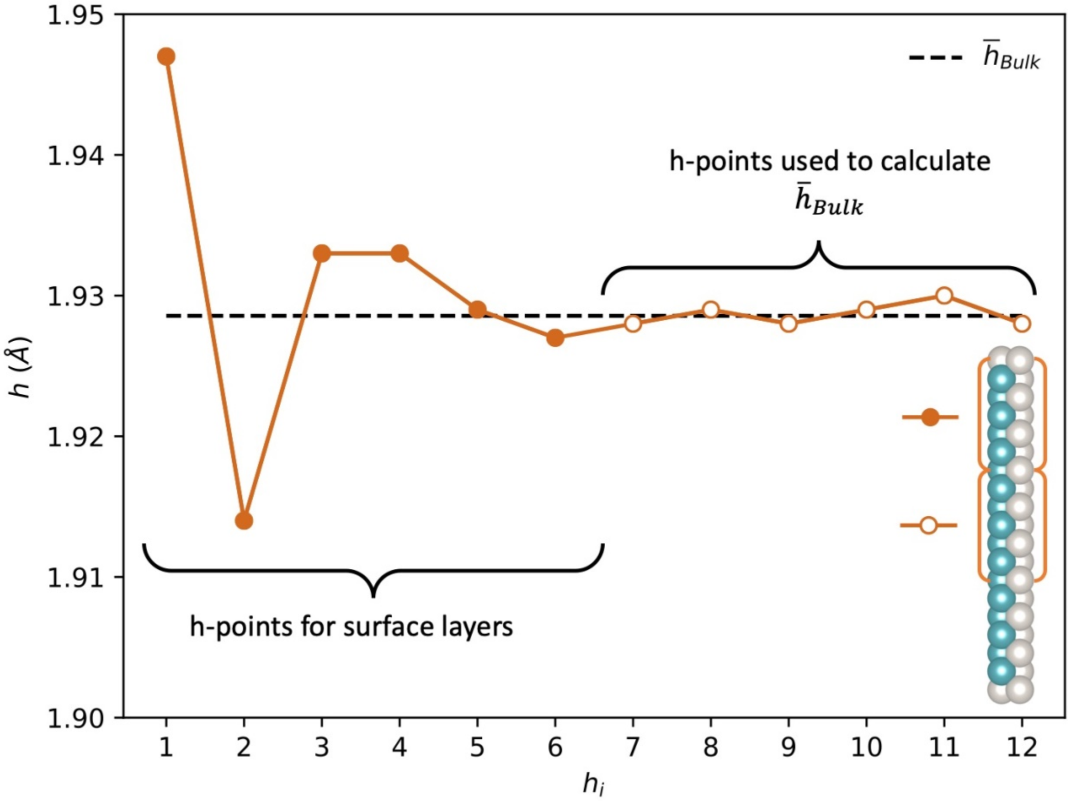

Fig. S13

Average spacing between different layers for the Pt-terminated 100-surface of the PtRh alloy. The upper-surface region and the bulk region are indicated. In this example, a $(\sqrt{2} \times \sqrt{2})$R45° unit cell with 19 layers is used to estimate h at the bulk limit. The number of spacing points (h_i) for the surface layers is 2/3 (upper and lower surfaces) of the total number of spacing points (n layers -1), and are not considered for the calculation of $\overline{h}_{\text{bulk}}$. An integer division is done for h-points that are not divisible by 3. Under these conditions, the surface layers are not exactly 2/3.

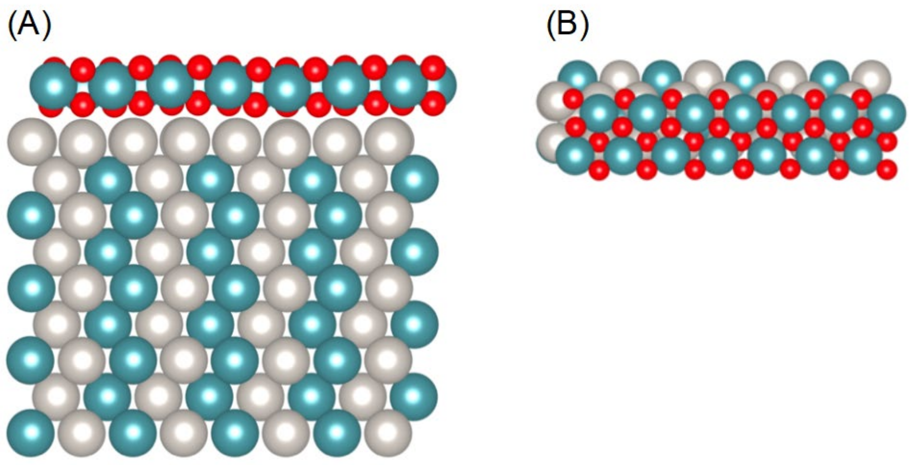

Fig. S14

Structural model for the surface oxide covered PtRh 100 surface. 100 facet of platinum and rhodium alloy top Pt-terminated with a RhO_2 overlayer in a 8×2 unit cell, side view (A) and top view (B).

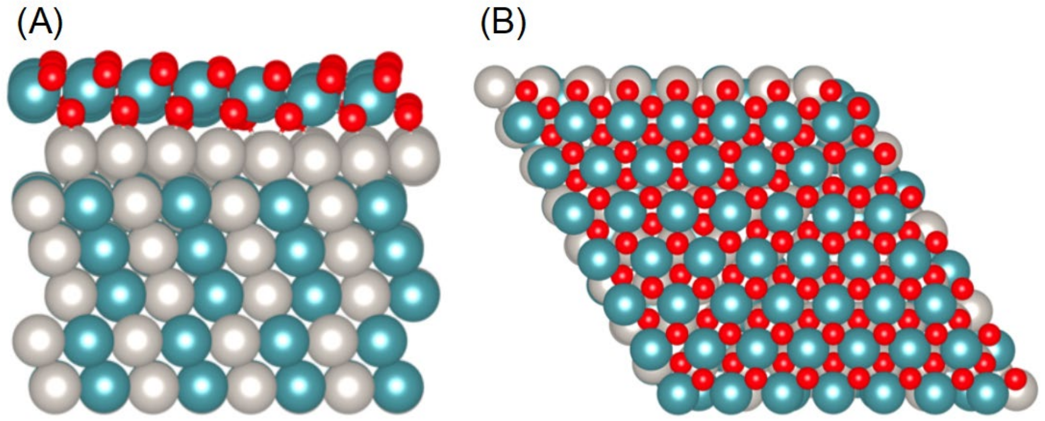

Fig. S15

Structural model for the surface oxide covered PtRh 111 surface. 111 facet of platinum and rhodium alloy top Pt-terminated with a RhO_2 overlayer in a 8x8 unit cell, side view (A) and top view (B).

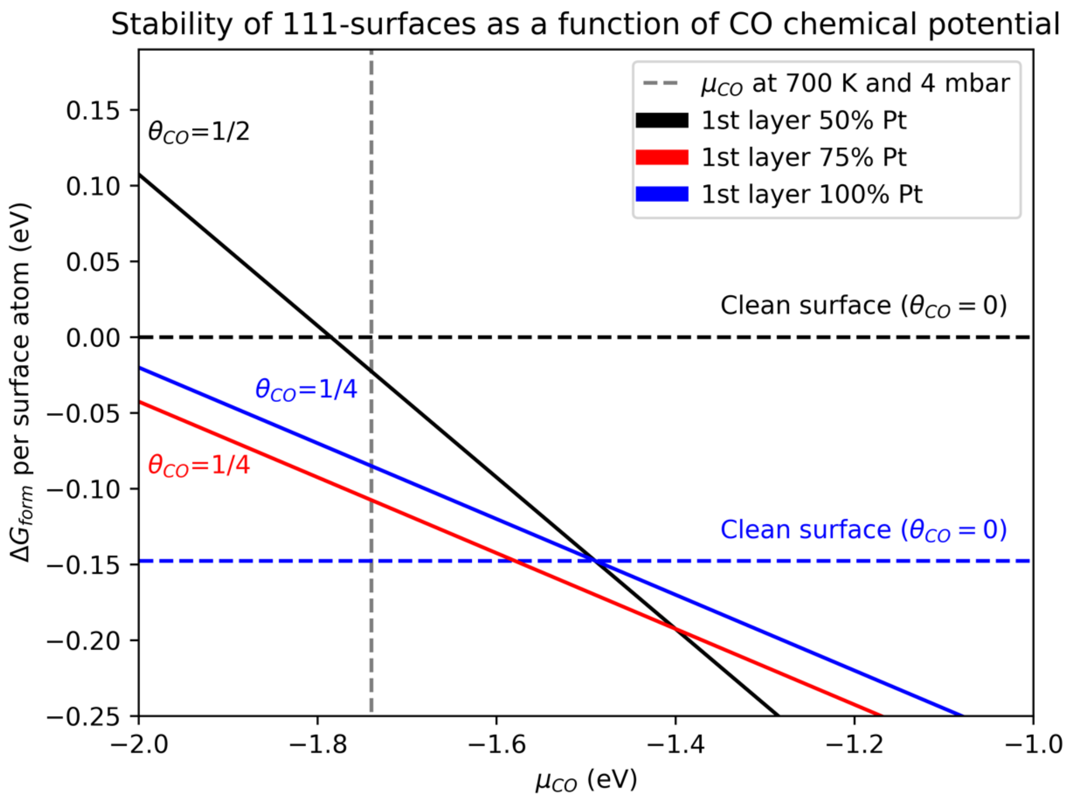

Fig. S16

Formation free energies ΔG_{form} of relevant 111-surfaces as a function of the chemical potential of CO. The chemical potential of CO corresponding to experimental conditions is indicated by a vertical dashed line. θ_{CO} is the CO coverage on a PtRh surface. Continuous and dashed lines are color coded as follows: Black lines - surfaces with a first layer composition of 50% Pt and 50% Rh; Red lines - surfaces with a first layer composition of 75% Pt and 25% Rh; Blue lines – surfaces with a first layer composition of 100% Pt. The composition of the 2nd and lower layers is 50% Pt and 50% Rh, regardless of the first layer composition.

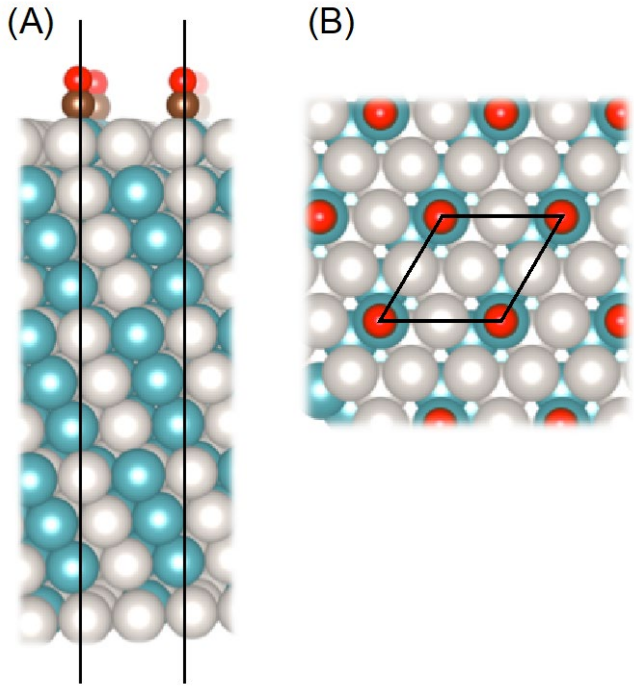

Fig. S17

Structural model for the CO covered PtRh 111 surface. 111 facet of the platinum and rhodium alloy with a quarter of CO coverage in a 2× 2 unit cell side view (A) and top view (B). The first layer is composed of 75% Pt and 25% Rh, whereas the subsequent layers are composed of 50% Pt and 50% Rh.

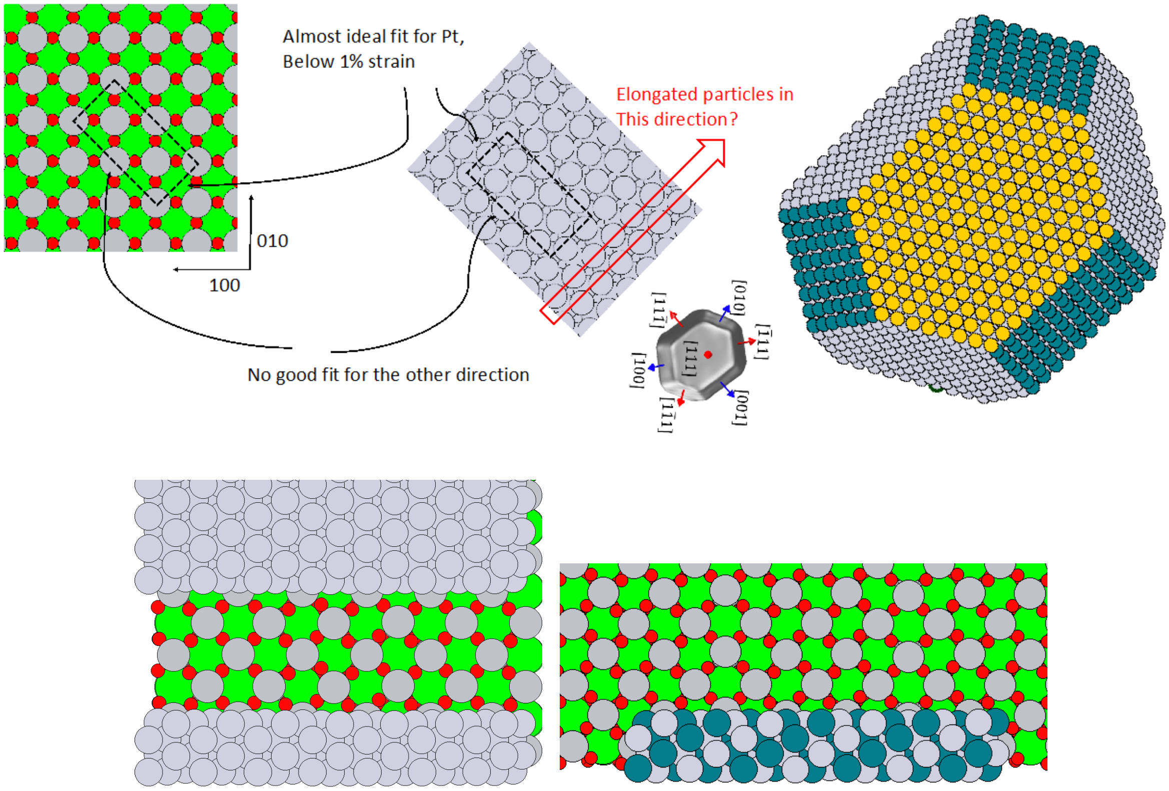

Fig. S18

Illustration of the oxide single crystal and metal alloy PtRh nanoparticle orientation for the construction of the interface. The theoretical aspect ratio height to diameter of 0.54 matches well the experimental value of 0.58. A slight elongation of the nanoparticle in its [110] direction is observed, lifting the threefold symmetry of the ideal Wulff shape shown in Fig. 1D. This may be due to a smaller lattice mismatch at the interface along the substrate [110] direction, leading to an anisotropic growth.

Tables S1-S12

Table S1

Gas environment before, during and after the *operando* CXDI experiment.

	Sample temperature (°K)	Ar Flow (ml min^{-1})	CO Flow (ml min^{-1})	O_2 Flow (ml min^{-1})	H_2 Flow (ml min^{-1})
Pre-Cleaning	623	48	0	0	2
Condition I (Ar)	700	50	0	0	0
Condition II (Ar+CO)	700	42	8	0	0
Condition III (Ar+CO+O_2)	700	38	8	4	0
Condition IV (Ar+CO)	700	42	8	0	0
Post-Treatment	700	48	0	0	2
Cooling down	25	50	0	0	0

Table S2

Range of dataset in reciprocal space (Q) and voxel size in real space.

Parameter	Ar (I)	Ar+CO (II)	Ar+CO+O_2 (III)	Ar+CO (IV)
Q_x [nm^{-1}]	±1.016	±0.589	±0.956	±0.956
Q_y [nm^{-1}]	±1.064	±1.062	±1.062	±1.062
Q_z [nm^{-1}]	±1.63	±1.451	±1.451	±1.451
Voxel size, x [nm]	3.1	5.3	3.3	3.3
Voxel size, y [nm]	3.0	3.0	3.0	3.0
Voxel size, z [nm]	1.9	2.2	2.2	2.2

Table S3

Spatial resolution calculated using the PRTF and the PSF for the reconstructed PtRh nanoparticle, Figure S12.

Parameter	Ar (I)	Ar+CO (II)	Ar+CO+O_2 (III)	Ar+CO (IV)
Resolution (PRTF), [nm]	14.8	12.1	14.8	18.0
Resolution (PSF), $[111], [\bar{1}\bar{1}\bar{1}]$ [nm]	4.1	4.0	5.9	5.5
Resolution (PSF), $[11\bar{1}], [\bar{1}\bar{1}1]$ [nm]	7.5	8.3	7.1	6.9
Resolution (PSF), $[1\bar{1}1], [\bar{1}1\bar{1}]$ [nm]	7.5	9.2	6.0	5.9
Resolution (PSF), $[\bar{1}11], [1\bar{1}\bar{1}]$ [nm]	7.6	7.8	7.0	6.7
Resolution (PSF), $[100]$ [nm]	6.8	6.5	6.3	6.1
Resolution (PSF), $[010]$ [nm]	6.1	7.4	7.8	7.4
Resolution (PSF), $[001]$ [nm]	6.7	7.1	6.0	6.1

Table S4

Overview of the theoretical PtRh alloy average strain values of a 100 facet in a $(\sqrt{2} \times \sqrt{2})R45°$ unit cell, reference structures are marked in bold. Oxygen adsorption is evaluated at 700 K and 2 mbar. [a] bridge position. [b] 4-fold position. The bulk composition (3rd layer and below) is fixed at 50% Pt.

Oxygen Coverage	1st layer	2nd layer	ϵ_{zz}^{t} (%)	ϵ_{zz}^{t*} ($\alpha = 54°$) (%)	Relative Stability/surf. atom(eV)
**0**	**50% Pt**	**50% Pt**	-0.14	**-0.05**	**0.00**
0	100% Pt	50% Pt	0.03	0.01	-0.15
0	100% Pt	100% Rh	-0.38	-0.13	-0.20
0	100% Rh	50% Pt	-0.32	-0.11	0.15
1/2[a]	100% Pt	50% Pt	0.29	0.10	-0.25
1/2[a]	100% Pt	100% Rh	-0.09	-0.03	-0.28
1/2[a]	50% Pt	50% Pt	0.14	0.05	-0.33
1/2[a]	100% Rh	50% Pt	-0.03	-0.01	-0.40
1/2[b]	100% Rh	50% Pt	0.06	0.02	-0.35
1[a]	100% Pt	50% Pt	0.43	0.15	0.06
1[a]	100% Pt	100% Rh	0.03	0.01	0.08
1[a]	50% Pt	50% Pt	0.35	0.12	-0.26
1[a]	100% Rh	50% Pt	0.26	0.09	-0.51

Table S5

Overview of the theoretical PtRh alloy average strain values of a 111 facet (2×2 unit cell), reference structures are marked in bold. Oxygen adsorption is evaluated at 700 K and 2 mbar. The bulk composition (3rd layer and below) is fixed at 50% Pt.

Oxygen Coverage	1st layer	2nd layer	ϵ_{zz}^{t} (%)	ϵ_{zz}^{t*} ($\alpha = 70°$) (%)	Relative Stability/surf. atom (eV)
**0**	**50% Pt**	**50% Pt**	**-0.06**	**-0.01**	**0.00**
0	100% Pt	50% Pt	0.23	0.03	-0.15
0	100% Pt	25% Pt	0.02	0.00	-0.16
0	100% Pt	100% Rh	-0.23	-0.03	-0.15
0	100% Rh	100% Pt	0.15	0.02	0.25
0	100% Rh	75% Pt	-0.06	-0.01	0.22
0	100% Rh	50% Pt	-0.28	-0.03	0.17
0	25% Pt	75% Pt	0.09	0.01	0.13
0	75% Pt	25% Pt	-0.13	-0.02	-0.09
1/4	50% Pt	50% Pt	0.21	0.02	-0.28
1/4	100% Pt	50% Pt	0.42	0.05	-0.17
1/4	100% Pt	25% Pt	0.15	0.02	-0.06
1/4	100% Pt	100% Rh	-0.08	-0.01	-0.16
1/4	100% Rh	100% Pt	0.46	0.05	-0.17
1/4	100% Rh	75% Pt	0.22	0.02	-0.15
1/4	100% Rh	50% Pt	0.02	0.00	-0.18
1/4	25% Pt	75% Pt	0.39	0.05	-0.11
1/4	75% Pt	25% Pt	0.09	0.01	-0.13
1/2	50% Pt	50% Pt	0.33	0.04	-0.42
1/2	100% Pt	50% Pt	0.44	0.05	-0.01
1/2	100% Pt	25% Pt	1.27	0.15	0.10
1/2	100% Pt	100% Rh	-0.06	-0.01	0.01
1/2	100% Rh	100% Pt	0.67	0.08	-0.45
1/2	100% Rh	50% Pt	0.22	0.03	-0.42
1/2	100% Rh	75% Pt	0.49	0.06	-0.35
1/2	75% Pt	25% Pt	0.78	0.09	0.05
1/2	25% Pt	75% Pt	0.61	0.07	-0.29
3/4	50% Pt	50% Pt	0.38	0.04	-0.24
3/4	100% Pt	50% Pt	0.31	0.04	0.35
3/4	100% Pt	25% Pt	1.17	0.14	0.56
3/4	100% Pt	100% Rh	-0.13	-0.01	0.35
3/4	100% Rh	100% Pt	0.78	0.09	-0.56
3/4	100% Rh	75% Pt	0.62	0.07	-0.38
3/4	100% Rh	50% Pt	0.33	0.04	-0.54
3/4	25% Pt	75% Pt	0.87	0.10	-0.19

3/4	75% Pt	25% Pt	0.55	0.06	0.36
1	50% Pt	50% Pt	0.38	0.04	0.10
1	100% Pt	50% Pt	0.11	0.01	0.87
1	100% Pt	25% Pt	1.34	0.16	0.88
1	100% Pt	100% Rh	-0.23	-0.03	0.86
1	100% Rh	100% Pt	0.86	0.10	-0.55
1	100% Rh	75% Pt	0.79	0.09	-0.32
1	100% Rh	50% Pt	0.45	0.05	-0.53
1	25% Pt	75% Pt	0.87	0.10	0.09
1	75% Pt	25% Pt	0.09	0.01	0.50

Table S6

Overview of the theoretical PtRh alloy average strain values of a 111 facet with a different number of layers used for the calculation. For both 111 and 100 facets 11 layer slabs were used. The bulk composition (3rd layer and below) is fixed at 50% Pt.

Number of Layers	1st layer	2nd layer	ϵ_{zz}^{t} (%)	ϵ_{zz}^{t*} ($\alpha = 70°$) (%)	Relative Stability/surf. atom (eV)
**9**	**50% Pt**	**50% Pt**	**-0.06**	**-0.01**	**0.00**
11	50% Pt	50% Pt	-0.06	-0.01	0.00
18	50% Pt	50% Pt	0.00	0.00	0.00
9	100% Pt	50% Pt	0.33	0.04	-0.15
11	100% Pt	50% Pt	0.23	0.03	-0.15
18	100% Pt	50% Pt	0.27	0.03	-0.15
9	100% Rh	50% Pt	-0.35	-0.04	0.18
11	100% Rh	50% Pt	-0.28	-0.03	0.17
18	100% Rh	50% Pt	-0.23	-0.03	0.19

Table S7

Comparison of different functionals in predicting strain values averaged over 2 nm thickness and stability. The surface composition below the first layer is 50% Pt and 50% Rh. Slabs are 9-layer thick.

Surface	Functionals	Oxygen Coverage	1st layer	ϵ_{zz}^{t*} ($\alpha = 54°$) (%)	Relative Stability/surf. atom (eV)
100	PBE	**0**	**50% Pt**	**-0.11**	**0.00**
		0	100% Pt	-0.03	-0.26
		0	100% Rh	-0.18	0.26
	PBE-D3	**0**	**50% Pt**	**-0.08**	**0.00**
		0	100% Pt	-0.01	-0.15
		0	100% Rh	-0.15	0.15
	BEEF-vdW	**0**	**50% Pt**	**-0.15**	**0.00**
		0	100% Pt	-0.06	-0.16
		0	100% Rh	-0.23	0.17

Surface	Functionals	Oxygen Coverage	1st layer	ϵ_{zz}^{t} (%)	ϵ_{zz}^{t*} ($\alpha = 70°$) (%)	Relative Stability/surf. atom (eV)
111	PBE	**0**	**50% Pt**	**-0.10**	**0.01**	**0.00**
		1/4	50% Pt	0.22	0.03	-0.29
		0	100% Pt	0.29	0.03	-0.25
		1/4	100% Pt	0.42	0.05	-0.29
		0	100% Rh	-0.37	-0.04	0.28
		3/4	100% Rh	0.33	0.04	-0.49
	PBE-D3	**0**	**50% Pt**	**-0.06**	**-0.01**	**0.00**
		1/4	50% Pt	0.24	0.03	-0.28
		0	100% Pt	0.33	0.04	-0.15
		1/4	100% Pt	0.47	0.06	-0.17
		0	100% Rh	-0.35	-0.04	0.18

		3/4	100% Rh	0.29	0.03	-0.54
	BEEF-vdW	**0**	**50% Pt**	**-0.17**	**-0.02**	**0.00**
		1/4	50% Pt	0.16	0.02	-0.25
		0	100% Pt	0.26	0.03	-0.17
		1/4	100% Pt	0.39	0.05	-0.16
		0	100% Rh	-0.48	-0.06	0.18
		3/4	100% Rh	0.21	0.02	-0.41

Table S8

Overview of the theoretical PtRh alloy average strain values on a 100 facet (2×8 unit cell) with a RhO_2 overlayer. Reference structures are marked in bold. The surface composition below the first and second layers are 50% Pt and 50% Rh. Formation free energies are referenced to the stoichiometric clean slabs (excluding RhO_2) and fcc(Rh) bulk and O_2. Since the number of layers on 6-layer slabs are not sufficient to estimate $\overline{h}_{\mathrm{bulk}}$ accurately, leading to inaccurate mean strain values, $\overline{h}_{\mathrm{bulk}}$ from 9-layer slabs was used instead. The mean strain is including both with and without considering the RhO_2 overlayer as a metallic layer contributing to the interlayer spacings.

Number of Layers	1st layer	2nd layer	ϵ_{zz}^{t*} ($\alpha = 54°$) (%) including RhO_2	ϵ_{zz}^{t*} ($\alpha = 54°$) (%) excluding RhO_2	Relative Stability per surface atom (eV)	Formation free energy per surface atom (eV)
**6**	**50% Pt**	**50% Pt**	**1.79**	**-0.03**	**0.00**	**-0.92**
6	100% Pt	50% Pt	2.01	0.04	0.01	-0.91
6	100% Rh	50% Pt	1.58	-0.14	-0.03	-0.95
6	100% Pt	100% Rh	1.93	-0.13	-0.02	-0.94
**9**	**50% Pt**	**50% Pt**	**1.77**	**-0.04**	**0.00**	**-0.93**
9	100% Pt	50% Pt	1.99	0.04	0.01	-0.92
9	100% Rh	50% Pt	1.58	-0.12	-0.03	-0.96
9	100% Pt	100% Rh	1.80	-0.15	-0.02	-0.95

Table S9

Overview of the theoretical PtRh alloy average strain values on a 111 facet (8×8×6 unit cell) with a RhO_2 overlayer. Reference structures are marked in bold. The surface composition below the first and second layers are 50% Pt and 50% Rh. Formation free energies are referenced to the stoichiometric clean slabs (excluding RhO_2) and fcc(Rh) bulk and O_2. Since the number of layers on 6-layer slabs are not sufficient to estimate $\overline{h}_{\text{bulk}}$ accurately, leading to inaccurate mean strain values, $\overline{h}_{\text{bulk}}$ from 9-layer slabs was used instead. The mean strain is including both with and without considering the RhO_2 overlayer as a metallic layer contributing to the interlayer spacings.

1st layer	2nd layer	ϵ_{zz}^{t} (%) including RhO_2	ϵ_{zz}^{t*} ($\alpha = 70°$) (%) including RhO_2	ϵ_{zz}^{t} (%) excluding RhO_2	ϵ_{zz}^{t*} ($\alpha = 70°$) (%) excluding RhO_2	Relative Stability per surface atom (eV)	Formation free energy per surface atom (eV)
**50% Pt**	**50% Pt**	**4.02**	**0.47**	**-0.02**	**0.00**	**0.00**	**-0.69**
100% Pt	50% Pt	4.67	0.55	0.22	0.03	0.02	-0.67
100% Rh	50% Pt	3.37	0.39	-0.27	-0.03	-0.02	-0.71
100% Pt	100% Rh	4.47	0.52	-0.35	-0.04	0.00	-0.69

Table S10

Overview of the theoretical PtRh alloy average strain values on a 100 facet in a $(\sqrt{2} \times \sqrt{2})$R45° unit cell. 11-layer slabs were used as models. Reference structures are marked in bold. CO adsorption is evaluated at 700 K and 4 mbar.

CO Coverage	1st layer	2nd layer	bulk	ϵ_{zz}^{t*} ($\alpha = 54°$) (%)	Relative Stability/surf. atom (eV)
**0**	**50% Pt**	**50% Pt**	**50% Pt**	**-0.05**	**0.00**
1/2	50% Pt	50% Pt	50% Pt	0.05	-0.02
1	50% Pt	50% Pt	50% Pt	0.17	0.32
0	100% Pt	50% Pt	50% Pt	0.01	-0.15
1/2	100% Pt	50% Pt	50% Pt	0.14	-0.14
1	100% Pt	50% Pt	50% Pt	0.26	0.46
0	100% Rh	50% Pt	50% Pt	-0.11	0.15
1/2	100% Rh	50% Pt	50% Pt	-0.04	0.10
1	100% Rh	50% Pt	50% Pt	0.06	0.35

Table S11

Overview of the theoretical PtRh alloy average strain values on a 111 facet (2×2 unit cell). 11-layer slabs were used as models. Reference structures are marked in bold. CO adsorption is evaluated at 700 K and 4 mbar.

CO Coverage	1st layer	2nd layer	bulk	ϵ_{zz}^{t} (%)	ϵ_{zz}^{t*} ($\alpha =$ 70°) (%)	Relative Stability/surf. atom (eV)
**0**	**50% Pt**	**50% Pt**	**50% Pt**	**-0.06**	**-0.01**	**0.00**
1/4	50% Pt	50% Pt	50% Pt	0.15	0.02	-0.05
1/2	50% Pt	50% Pt	50% Pt	0.44	0.05	-0.02
3/4	50% Pt	50% Pt	50% Pt	0.68	0.08	0.15
1	50% Pt	50% Pt	50% Pt	0.51	0.06	0.78
0	100% Pt	50% Pt	50% Pt	0.23	0.03	-0.15
1/4	100% Pt	50% Pt	50% Pt	0.53	0.06	-0.09
1/2	100% Pt	50% Pt	50% Pt	0.59	0.07	0.04
3/4	100% Pt	50% Pt	50% Pt	0.70	0.08	0.27
1	100% Pt	50% Pt	50% Pt	0.58	0.07	0.99
0	100% Rh	50% Pt	50% Pt	-0.28	-0.03	0.17
1/4	100% Rh	50% Pt	50% Pt	-0.06	-0.01	0.11
1/2	100% Rh	50% Pt	50% Pt	0.14	0.02	0.08
3/4	100% Rh	50% Pt	50% Pt	0.24	0.03	0.13
1	100% Rh	50% Pt	50% Pt	0.53	0.06	0.58
0	75% Pt	50% Pt	50% Pt	0.09	0.01	-0.08
1/4	75% Pt	50% Pt	50% Pt	0.31	0.04	-0.11
0	25% Pt	50% Pt	50% Pt	-0.18	-0.02	0.09
1/4	25% Pt	50% Pt	50% Pt	0.06	0.01	0.04

Table S12

Overview of the theoretical PtRh alloy average strain values on a 111 facet (2×7$\sqrt{2}$ unit cell) including the STO support. Reference structures are marked in bold. The surface composition beyond the first and second layers are 50% Pt and 50% Rh.

Number of Layers	1st layer	2nd layer	ϵ_{zz}^{t} (%)	Relative Stability per surface atom (eV)
**9**	**50% Pt**	**50% Pt**	**0.05**	**0.00**
9	100% Pt	50% Pt	0.28	-0.01
9	100% Rh	50% Pt	-0.19	0.03

REFERENCES AND NOTES

1. G. Ertl, H. Knözinger, F. Schüth, J. Weitkamp, *Handbook of Heterogeneous Catalysis: Online* (Wiley-VCH, 2008).
2. K. F. Kalz, R. Kraehnert, M. Dvoyashkin, R. Dittmeyer, R. Gläser, U. Krewer, K. Reuter, J.-D. Grunwaldt, Future challenges in heterogeneous catalysis: Understanding catalysts under dynamic reaction conditions. *ChemCatChem* **9**, 17–29 (2017).
3. O. Deutschmann, H. Knözinger, K. Kochloefl, T. Turek, in *Ullmann's Encyclopedia of Industrial Chemistry* (Wiley-VCH, 2011).
4. B. Roldan Cuenya, F. Behafarid, Nanocatalysis: Size- and shape-dependent chemisorption and catalytic reactivity. *Surf. Sci. Rep.* **70**, 135–187 (2015).
5. S. B. Vendelbo, C. F. Elkjær, H. Falsig, I. Puspitasari, P. Dona, L. Mele, B. Morana, B. J. Nelissen, R. van Rijn, J. F. Creemer, P. J. Kooyman, S. Helveg, Visualization of oscillatory behaviour of Pt nanoparticles catalysing CO oxidation. *Nat. Mater.* **13**, 884–890 (2014).
6. H. Yoshida, Y. Kuwauchi, J. R. Jinschek, K. Sun, S. Tanaka, M. Kohyama, S. Shimada, M. Haruta, S. Takeda, Visualizing gas molecules interacting with supported nanoparticulate catalysts at reaction conditions. *Science* **335**, 317–319 (2012).
7. T. Avanesian, S. Dai, M. J. Kale, G. W. Graham, X. Pan, P. Christopher, Quantitative and atomic-scale view of CO-induced Pt nanoparticle surface reconstruction at saturation coverage via DFT calculations coupled with *in situ* TEM and IR. *J. Am. Chem. Soc.* **139**, 4551–4558 (2017).
8. F. Tao, P. A. Crozier, Atomic-scale observations of catalyst structures under reaction conditions and during catalysis. *Chem. Rev*. **116**, 3487–3539 (2016).
9. J. Miao, T. Ishikawa, I. K. Robinson, M. M. Murnane, Beyond crystallography: Diffractive imaging using coherent x-ray light sources. *Science* **348**, 530–535 (2015).
10 I. Robinson, R. Harder, Coherent x-ray diffraction imaging of strain at the nanoscale. *Nat. Mater.* **8**, 291–298 (2009).
11. I. A. Vartanyants, I. K. Robinson, Partial coherence effects on the imaging of small crystals using coherent x-ray diffraction. *J. Phys. Condens. Matter* **13**, 10593–10611 (2001).
12. A. Ulvestad, M. J. Welland, W. Cha, Y. Liu, J. W. Kim, R. Harder, E. Maxey, J. N. Clark, M. J. Highland, H. You, P. Zapol, S. O. Hruszkewycz, G. B. Stephenson, Three-dimensional imaging of dislocation dynamics during the hydriding phase transformation. *Nat. Mater.* **16**, 565–571 (2017).

13. D. Kim, M. Chung, S. Kim, K. Yun, W. Cha, R. Harder, H. Kim, Defect dynamics at a single Pt nanoparticle during catalytic oxidation. *Nano Lett.* **19**, 5044–5052 (2019).
14. D. Kim, M. Chung, J. Carnis, S. Kim, K. Yun, J. Kang, W. Cha, M. J. Cherukara, E. Maxey, R. Harder, K. Sasikumar, S. K. R. S. Sankaranarayanan, A. Zozulya, M. Sprung, D. Riu, H. Kim, Active site localization of methane oxidation on Pt nanocrystals. *Nat. Commun.* **9**, 3422 (2018).
15. S. Fernández, L. Gao, J. P. Hofmann, J. Carnis, S. Labat, G. A. Chahine, A. J. F. van Hoof, M. W. G. M. Verhoeven, T. U. Schülli, E. J. M. Hensen, O. Thomas, M.-I. Richard, *In situ* structural evolution of single particle model catalysts under ambient pressure reaction conditions. *Nanoscale* **11**, 331–338 (2019).
16. M. Abuin, Y. Y. Kim, H. Runge, S. Kulkarni, S. Maier, D. Dzhigaev, S. Lazarev, L. Gelisio, C. Seitz, M.-I. Richard, T. Zhou, V. Vonk, T. F. Keller, I. A. Vartanyants, A. Stierle, Coherent x-ray imaging of CO-adsorption-induced structural changes in Pt nanoparticles: Implications for catalysis. *ACS Appl. Nano Mater.* **2**, 4818–4824 (2019).
17. A. R. Passos, A. Rochet, L. M. Manente, A. F. Suzana, R. Harder, W. Cha, F. Meneau, Three-dimensional strain dynamics govern the hysteresis in heterogeneous catalysis. *Nat. Commun.* **11**, 4733 (2020).
18. A. Ulvestad, K. Sasikumar, J. W. Kim, R. Harder, E. Maxey, J. N. Clark, B. Narayanan, S. A. Deshmukh, N. Ferrier, P. Mulvaney, S. K. R. S. Sankaranarayanan, O. G. Shpyrko, In situ 3D imaging of catalysis induced strain in gold nanoparticles. *J. Phys. Chem. Lett.* **7**, 3008–3013 (2016).
19. J. Y. Park, Y. Zhang, M. Grass, T. Zhang, G. A. Somorjai, Tuning of catalytic CO oxidation by changing composition of Rh−Pt bimetallic nanoparticles. *Nano Lett*. **8**, 673–677 (2008).
20. U. Hejral, D. Franz, S. Volkov, S. Francoual, J. Strempfer, A. Stierle, Identification of a catalytically highly active surface phase for CO oxidation over PtRh nanoparticles under operando reaction conditions. *Phys. Rev. Lett.* **120**, 126101 (2018).
21. S. B. Maisel, T. C. Kerscher, S. Müller, No miscibility gap in Pt–Rh binary alloys: A first-principles study. *Acta Mater.* **60**, 1093–1098 (2012).
22. W. L. Winterbottom, Equilibrium shape of a small particle in contact with a foreign substrate. *Acta Metall.* **15**, 303–310 (1967).
23. T. Kawaguchi, T. F. Keller, H. Runge, L. Gelisio, C. Seitz, Y. Y. Kim, E. R. Maxey, W. Cha, A. Ulvestad, S. O. Hruszkewycz, R. Harder, I. A. Vartanyants, A. Stierle, H. You, Gas-induced

segregation in Pt-Rh alloy nanoparticles observed by *in situ* Bragg coherent diffraction imaging. *Phys. Rev. Lett.* **123**, 246001 (2019).

24. J. P. Perdew, K. Burke, M. Ernzerhof, Generalized gradient approximation made simple. *Phys. Rev. Lett.* **77**, 3865–3868 (1996).
25. E. Platzgummer, M. Sporn, R. Koller, S. Forsthuber, M. Schmid, W. Hofer, P. Varga, Temperature-dependent segregation on Pt25Rh75(111) and (100). *Surf. Sci.* **419**, 236–248 (1999).
26. A. V. Ruban, H. L. Skriver, Calculated surface segregation in transition metal alloys. *Comput. Mater. Sci.* **15**, 119–143 (1999).
27. K. Yuge, A. Seko, A. Kuwabara, F. Oba, I. Tanaka, First-principles study of bulk ordering and surface segregation in Pt-Rh binary alloys. *Phys. Rev. B* **74**, 174202 (2006).
28. G. Ertl, M. Neumann, K. M. Streit, Chemisorption of CO on the Pt(111) surface. *Surf. Sci.* **64**, 393–410 (1977).
29. J. Wellendorff, T. L. Silbaugh, D. Garcia-Pintos, J. K. Nørskov, T. Bligaard, F. Studt, C. T. Campbell, A benchmark database for adsorption bond energies to transition metal surfaces and comparison to selected DFT functionals. *Surf. Sci.* **640**, 36–44 (2015).
30. M. Jørgensen, H. Grönbeck, The site-assembly determines catalytic activity of nanoparticles. *Angew. Chem. Int. Ed.* **57**, 5086–5089 (2018).
31. R. Westerström, J. G. Wang, M. D. Ackermann, J. Gustafson, A. Resta, A. Mikkelsen, J. N. Andersen, E. Lundgren, O. Balmes, X. Torrelles, J. W. M. Frenken, B. Hammer, Structure and reactivity of a model catalyst alloy under realistic conditions. *J. Phys. Condens. Matter* **20**, 184018 (2008).
32. P. Nolte, A. Stierle, N. Y. Jin-Phillipp, N. Kasper, T. U. Schulli, H. Dosch, Shape changes of supported Rh nanoparticles during oxidation and reduction cycles. *Science* **321**, 1654–1658 (2008).
33. M. Sporn, E. Platzgummer, E. L. D. Gruber, M. Schmid, W. Hofer, P. Varga, A quantitative LEED analysis of the oxygen-induced p(3×1) reconstruction of $Pt_{25}Rh_{75}$(100). *Surf. Sci.* **416**, 384–395 (1998).
34. G. Koster, B. L. Kropman, G. J. H. M. Rijnders, D. H. A. Blank, H. Rogalla, Quasi-ideal strontium titanate crystal surfaces through formation of strontium hydroxide. *Appl. Phys. Lett*. **73**, 2920–2922 (1998).
35. A. Stierle, T. F. Keller, H. Noei, V. Vonk, R. Roehlsberger, DESY NanoLab. *JLSRF* **2**, A76 (2016).

36. S. J. Leake, G. A. Chahine, H. Djazouli, T. Zhou, C. Richter, J. Hilhorst, L. Petit, M.-I. Richard, C. Morawe, R. Barrett, L. Zhang, R. A. Homs-Regojo, V. Favre-Nicolin, P. Boesecke, T. U. Schülli, The Nanodiffraction beamline ID01/ESRF: A microscope for imaging strain and structure. *J. Synchrotron Radiat.* **26**, 571–584 (2019).
37. M. A. Pfeifer, G. J. Williams, I. A. Vartanyants, R. Harder, I. K. Robinson, Three-dimensional mapping of a deformation field inside a nanocrystal. *Nature* **442**, 63–66 (2006).
38. J. R. Fienup, Phase retrieval algorithms: A personal tour [Invited]. *Appl. Opt.* **52**, 45–56 (2013).
39. J. R. Fienup, Phase retrieval algorithms: A comparison. *Appl. Opt.* **21**, 2758–2769 (1982).
40. S. Marchesini, H. He, H. N. Chapman, S. P. Hau-Riege, A. Noy, M. R. Howells, U. Weierstall, J. C. H. Spence, X-ray image reconstruction from a diffraction pattern alone. *Phys. Rev. B* **68**, 140101 (2003).
41. M. Guizar-Sicairos, S. T. Thurman, J. R. Fienup, Efficient subpixel image registration algorithms. *Opt. Lett*. **33**, 156–158 (2008).
42. G. Kresse, J. Furthmüller, Efficient iterative schemes for ab initio total-energy calculations using a plane-wave basis set. *Phys. Rev. B* **54**, 11169–11186 (1996).
43. G. Kresse, D. Joubert, From ultrasoft pseudopotentials to the projector augmented-wave method. *Phys. Rev. B* **59**, 1758–1775 (1999).
44. S. Grimme, J. Antony, S. Ehrlich, H. Krieg, A consistent and accurate *ab initio* parametrization of density functional dispersion correction (DFT-D) for the 94 elements H-Pu. *J. Chem. Phys.* **132**, 154104 (2010).
45. V. Komanicky, H. Iddir, K.-C. Chang, A. Menzel, G. Karapetrov, D. Hennessy, P. Zapol, H. You, Shape-dependent activity of platinum array catalyst. *J. Am. Chem. Soc*. **131**, 5732–5733 (2009).
46. J. W. Arblaster, Crystallographic properties of platinum. *Platin. Met. Rev.* **41**, 12–21 (1997).
47. J. W. Arblaster, Crystallographic properties of rhodium. *Platin. Met. Rev.* **41**, 184–189 (1997).
48. H. N. Chapman, A. Barty, S. Marchesini, A. Noy, S. P. Hau-Riege, C. Cui, M. R. Howells, R. Rosen, H. He, J. C. H. Spence, U. Weierstall, T. Beetz, C. Jacobsen, D. Shapiro, High-resolution ab initio three-dimensional x-ray diffraction microscopy. *J. Opt. Soc. Am. A* **23**, 1179 (2006).
49. M. J. Cherukara, W. Cha, R. J. Harder, Anisotropic nano-scale resolution in 3D Bragg coherent diffraction imaging. *Appl. Phys. Lett.* **113**, 203101 (2018).
50. D. S. C. Biggs, M. Andrews, Acceleration of iterative image restoration algorithms. *Appl. Opt.* **36**, 1766–1775 (1997).

51. P. A. Jansson, *Deconvolution of Images and Spectra* (Academic Press, ed. 2, 1997).
52. R. Grothausmann, S. Fiechter, R. Beare, G. Lehmann, H. Kropf, G. S. Vinod Kumar, I. Manke, J. Banhart, Automated quantitative 3D analysis of faceting of particles in tomographic datasets. *Ultramicroscopy* **122**, 65–75 (2012).
53. T. Lewiner, H. Lopes, A. W. Vieira, G. Tavares, Efficient implementation of marching cubes' cases with topological guarantees. *J. Graph. Tools* **8**, 1–11 (2003).
54. G. Taubin, T. Zhang, G. Golub, in *Computer Vision—ECCV '96*, B. Buxton, R. Cipolla, Eds. (Springer, 1996), vol. 1064, pp. 283–292.
55. M.-I. Richard, S. Fernández, J. Eymery, J. P. Hofmann, L. Gao, J. Carnis, S. Labat, V. Favre-Nicolin, E. J. M. Hensen, O. Thomas, T. U. Schülli, S. J. Leake, Crystallographic orientation of facets and planar defects in functional nanostructures elucidated by nano-focused coherent diffractive x-ray imaging. *Nanoscale* **10**, 4833–4840 (2018).
56. J. Carnis, S. Leake, The BCDI Python package can be downloaded from PyPi (https://pypi.org/project/bcdi/) or GitHub (https://github.com/carnisj/bcdi) (available at https://doi.org/10.5281/zenodo.3257616). carnisj/bcdi: V0.0.9 (Zenodo, 2020; https://zenodo.org/record/3257616).
57. J. Carnis, L. Gao, S. Fernández, G. Chahine, T. U. Schülli, S. Labat, E. J. M. Hensen, O. Thomas, J. P. Hofmann, M.-I. Richard, Facet-dependent strain determination in electrochemically synthetized platinum model catalytic nanoparticles, *Small* **17**, 2007702 (2021).
58. J. Gustafson, A. Mikkelsen, M. Borg, J. N. Andersen, E. Lundgren, C. Klein, W. Hofer, M. Schmid, P. Varga, L. Köhler, G. Kresse, N. Kasper, A. Stierle, H. Dosch, Structure of a thin oxide film on Rh(100). *Phys. Rev. B* **71**, 115442 (2005).
59. T. L. Silbaugh, C. T. Campbell, Energies of formation reactions measured for adsorbates on late transition metal surfaces. *J. Phys. Chem. C* **120**, 25161–25172 (2016).
